# Impact of Generalized Order Statistics and Its Dual on Entropy Estimation for the Exponentiated Generalized Pham Distribution: Theory and Applications

**DOI:** 10.3390/e28080909

**Published:** 2026-08-13

**Authors:** Zakiah I. Kalantan, Sulafah M. S. Binhimd, Asmaa M. Abd AL-Fattah, Asmaa A. Ahmed, Gannat R. AL-Dayian, Abeer A. EL-Helbawy, Mervat K. Abd Elaal

**Affiliations:** 1 Department of Statistics, Faculty of Sciences, King Abdulaziz University, Jeddah 21589, Saudi Arabia; 2 Department of Statistics, Faculty of Commerce, Al-Azhar University (Girls’ Branch), Cairo 11651, Egypt; 3 Department of Basic Sciences, Badr Institute of Science and Technology (BIS), Badr 11829, Egypt; 4 Higher Institute of Marketing, Commerce & Information Systems (MCI), Cairo 11856, Egypt

**Keywords:** exponentiated generalized distributions, entropy measures, Vtub-shaped lifetime distribution, generalized order statistics, maximum likelihood estimation

## Abstract

Entropy is a fundamental measure of uncertainty in reliability and lifetime analysis, and the structure of the observed data intrinsically influences its estimation. In many practical applications, inference relies on ordered or record-based samples, for which generalized order statistics and their dual form provide a comprehensive and unifying framework encompassing order statistics, reversed order statistics, and record values as special cases. Despite substantial progress in entropy estimation and lifetime modeling, little attention has been devoted to a unified entropy estimation framework for flexible lifetime models under generalized ordered sampling schemes. This paper investigates the impact of generalized order statistics and their dual form on the estimation of entropy measures for the exponentiated generalized Pham distribution. The proposed distribution extends the classical Pham model through additional shape flexibility, enabling it to accommodate diverse reliability behaviors and heterogeneous tail characteristics. Several fundamental properties are derived, and maximum likelihood estimation and corresponding confidence intervals for model parameters and entropy measures are developed under both generalized order statistics and dual generalized order statistics frameworks. The general results are further specialized to order statistics, reversed order statistics, and upper and lower record values. Applications to two real datasets demonstrate that the proposed distribution provides an excellent fit compared with competing models, as confirmed by goodness-of-fit measures. The findings underscore the pivotal structural role of generalized order statistics and their dual form in entropy-based inference, particularly for record or partially observed data, where the sampling design critically affects uncertainty quantification.

## 1. Introduction

In information theory, entropy is a fundamental measure of uncertainty or randomness in a system or probability distribution. It quantifies the expected amount of information conveyed by a random variable, thereby reflecting the degree of disorder or unpredictability inherent in the data. Measuring entropy has broad applications across diverse scientific and practical domains, including statistics, physics, chemistry, economics, insurance, financial modeling, and biological systems. For instance, in physics, entropy quantifies the disorder of thermodynamic systems, while in chemistry, it reflects the spontaneity of reactions. In economics and finance, it measures market uncertainty and portfolio diversification; in insurance, it assists in assessing risk variability; and in biology, it evaluates genetic diversity and physiological complexity. In statistics, entropy functions as an effective tool for model selection and the quantification of information content. A higher entropy value corresponds to a greater degree of uncertainty, implying a reduction in the amount of information that can be derived from each observation. Within the framework of statistical modeling and reliability analysis, entropy constitutes a vital analytical instrument for quantifying uncertainty and assessing system variability. Specifically, in lifetime data analysis, entropy provides an informative index for evaluating the randomness and unpredictability associated with the underlying life durations of components or systems.

Shannon entropy was introduced by [1] for a continuous random variable X, with *probability density function* (pdf), f(x), the Shannon entropy is mathematically defined as

(1) $$HS\left(f\right)=-\int_{-\infty }^{\infty }f\left(x\right)\ln\left[f\left(x\right)\right]dx.$$

Since the pioneering contribution of Shannon, several generalized entropy measures have been developed to provide broader perspectives on uncertainty characterization. Among these, the Rényi entropy introduced by Ref. [2] is one of the most prominent extensions, encompassing Shannon entropy as a limiting case and offering greater flexibility in analyzing the diversity and concentration properties of probability distributions. For a random variable X with pdf, f(x), the Rényi entropy is defined by:

(2) $$HR_{p}(f)=\frac{1}{1-p}\ln\left[\int_{-\infty }^{\infty }{\left[f\left(x\right)\right]}^{p}dx\right],\;p>0,p\neq 1,$$

which reduces to the Shannon entropy as p→1. The Rényi measure provides a flexible framework for emphasizing different regions of the probability distribution, depending on the value of p.

Ref. [3] generalized Shannon entropy into a one-parameter form based on the multifractal scaling of a quantity.

(3) $$HT_{q}\left(f\right)=\frac{1}{q-1}\left\{1-\int_{-\infty }^{\infty }{\left[f\left(x\right)\right]}^{q}dx\right\},\;q>0,q\neq 1,$$

where q(≠1)>0. Similar to Shannon entropy for continuous cases, this measure can also exhibit negative values. Shannon entropy is a particular case of Tsallis entropy; as q goes to 1, Tsallis entropy tends to Shannon entropy.

In addition to the theoretical development of entropy measures, considerable attention has been devoted to their statistical estimation. Ref. [4] established uniform-in-bandwidth consistency for kernel-type estimators of Shannon differential entropy. Ref. [5] investigated entropy estimation for several exponential distributions. Ref. [6] developed entropy estimators for the Weibull distribution based on record values, while [7] studied entropy measures for the Kumaraswamy distribution. Ref. [8] considered the estimation of Lomax entropy using *upper record* (UR) values under both informative and non-informative Bayesian priors. Ref. [9] proposed *maximum likelihood* (ML) and Bayesian estimation procedures for the Shannon and Rényi entropies of the inverse Weibull distribution. Ref. [10] introduced a maximum entropy approach for estimating the parameters of the Kumaraswamy distribution under moment constraints. More recently, Ref. [11] proposed a model integrating the Chen distribution with entropy transformation techniques to improve longevity data analysis in engineering and medical applications.

Although entropy estimation for lifetime distributions has been extensively investigated, most studies have considered only specific probability models or sampling schemes. Since practical reliability and survival studies frequently involve ordered rather than complete random samples, developing entropy estimation within unified ordered sampling frameworks remains an important and practically relevant research problem.

Several ordered sampling schemes have been developed for analyzing ordered observations, including *ordinary order statistics* (OS), UR values, Pfeifer records, and sequential order statistics. Although each has its own theoretical advantages, studying them separately often results in repeated mathematical developments and limits the generality of statistical inference.

To overcome these limitations, Ref. [12] introduced *generalized order statistics* (GOS), a unified framework for analyzing increasing ordered random variables. With the appropriate choice of its parameters, GOS includes OS, sequential order statistics, UR values, and Pfeifer records as special cases. Owing to its flexibility, GOS has become widely used in reliability, lifetime and survival analyses, engineering, hydrology, medicine, and other fields involving ordered observations.

Many practical applications, however, involve decreasingly ordered observations, such as longest component lifetimes, descending environmental measurements, and successive minimum observations. To address this limitation, Ref. [13] introduced *dual generalized order statistics* (DGOS), extending GOS to decreasingly ordered data. Ref. [14] subsequently established its theoretical foundations, showing that DGOS unifies *reversed order statistics* (ROS), *lower record* (LR) values, and lower Pfeifer records.

DGOS is generally more convenient when the *cumulative distribution function* (cdf), F(x), has a simple closed form, whereas GOS is preferable when the *survival function* (sf), 1−F(x), is more tractable. Thus, the two frameworks complement each other and extend the range of lifetime models that can be analyzed. Further developments and applications of DGOS are given by [15,16,17,18,19,20,21,22].

Record values are important special cases of GOS and DGOS that arise naturally when only successive extreme observations are available. They occur in numerous applications, including sporting records, meteorology, hydrology, reliability, finance, and economics. Successive maximum and minimum observations are referred to as UR and LR values, respectively. Because complete samples are often unavailable, record values provide an efficient basis for statistical inference in reliability and lifetime analyses. Additional applications of LR values are discussed in [23,24].

Let$\;X_{\left(1,n,m,k\right)},\;X_{\left(2,n,m,k\right)},\;{\ldots \;X}_{\left(n,n,m,k\right)}\;$denote n GOS, where k≥1 and m∈R. According to [12], their joint pdf is given by

(4) $$f_{X_{\left(1,n,m,k\right)},\;X_{\left(2,n,m,k\right)},\;{\cdots \;X}_{\left(n,n,m,k\right)}}\left(x_{\left(1\right)},\ldots ,x_{\left(n\right)}\right)=k\;\left(\prod_{j=1}^{n-1}\gamma_{j}\right)\left[\prod_{i=1}^{n-1}{\left(1-F\left(x_{\left(i\right)}\right)\right)}^{m}f\left(x_{\left(i\right)}\right)\right]\;\times {\left(1-F\left(x_{\left(n\right)}\right)\right)}^{k-1}f\left(x_{\left(n\right)}\right),$$

where$\;F^{-1}\left(0\right)<x_{\left(1\right)}\leq \cdots \leq x_{\left(n\right)}<F^{-1}(1)$, $\zeta_{r-1}=\prod_{j=1}^{r}\gamma_{j},\;r=1,\ldots n\;,\;\gamma_{j}=k+\left(n-j\right)\left(m+1\right),\;and\;\gamma_{n}=k.$

As special cases of GOS, setting m=0 and k=1 yields OS, whereas setting m=−1 and k=1 yields UR values.

On the other hand, let $T_{\left(1,n,m,k\right)},\;T_{\left(2,n,m,k\right)},\;{\ldots T}_{\left(n,n,m,k\right)}$ denote n DGOS. According to [14], the joint pdf has the form

(5) $$\begin{array}{ll} f_{T_{\left(1,n,m,k\right)},\;T_{\left(2,n,m,k\right)},\;{\ldots \;T}_{\left(n,n,m,k\right)}}^{*}\left(t_{\left(1\right)},\ldots ,t_{\left(n\right)}\right)= & k\;\left(\prod_{j=1}^{n-1}\gamma_{j}\right)\left[\prod_{i=1}^{n-1}{\left(F\left(t_{\left(i\right)}\right)\right)}^{m}f\left(t_{\left(i\right)}\right)\right]\\ & \times {\left(F\left(t_{\left(n\right)}\right)\right)}^{k-1}f\left(t_{\left(n\right)}\right)\;,\; \end{array}$$

where ${\;F}^{-1}\left(1\right)\geq x_{\left(1\right)}\ldots \geq x_{\left(n\right)}\geq F^{-1}\left(0\right)$,$\;n\in N,\;k\geq 1,\;m_{1},\ldots ,\;m_{n-1}=m,m\in R$ are the parameters such that$\;{\;\gamma }_{r}=k+\left(n-r\right)\left(m+1\right)\geq 1\;,\;\mathrm{for}\;\mathrm{all}\;1\leq r\leq n.$

As special cases of DGOS, setting m=0 and k=1 yields ROS, whereas setting m=−1 and k=1 yields LR values.

Together, GOS and DGOS provide a unified framework for increasing and decreasing ordered observations. This framework facilitates unified entropy estimation under multiple ordered sampling schemes without requiring separate mathematical derivations for each case.

While GOS and DGOS provide a unified framework for ordered observations, entropy estimation also depends on the underlying lifetime distribution. Therefore, selecting a flexible lifetime distribution capable of representing diverse reliability behaviors is equally important. In reliability engineering and software reliability, flexible hazard rate functions are essential for modeling complex failure patterns that cannot be adequately captured by classical distributions. Among the lifetime distributions proposed over the past three decades, the *Pham* (P) distribution has attracted considerable attention because of its Vtub-shaped *hazard rate function* (hrf) and its applicability to software reliability analysis.

The P distribution, introduced by Ref. [25] and known as the log–log distribution, possesses a Vtub-shaped hrf suitable for software reliability modeling. This pattern, characterized by a failure rate that initially rises slowly after the newborn mortality period and subsequently increases because of aging-related failures, is frequently observed in engineering and software reliability applications. The cdf and pdf of the P distribution are given, respectively, by

(6) $$G\left(x;\alpha ,\beta \right)=1-e^{\left(1-\beta^{x^{\alpha }}\right)},\;\alpha >0,\beta >1,x>0,$$

and

(7) $$g\left(x;\alpha ,\beta \right)=\alpha \ln\left(\beta \right)\;x^{\alpha -1}\beta^{x^{\alpha }}e^{1-\beta^{x^{\alpha }}},\alpha >0,\beta >1,x>0.$$

Ref. [25] demonstrated the applicability of the distribution by modeling helicopter component failure data. Subsequently, Ref. [26] summarized several theoretical properties of the distribution, Ref. [27] investigated Bayesian estimation of its parameters, Ref. [28] proposed software reliability models based on the distribution, Ref. [29] compared P-based software reliability models under testing coverage and operating-environment uncertainty, Ref. [30] introduced the unit P distribution, Ref. [31] proposed the extended P distribution, and Ref. [32] developed the inverse P distribution.

Despite these developments, the classical P distribution and its extensions may still be inadequate for modeling highly heterogeneous lifetime data with varying skewness and hazard rate behaviors. A common strategy for enhancing flexibility is to embed the baseline distribution within a more general family by introducing additional shape parameters, thereby improving flexibility while preserving mathematical tractability.

Ref. [33] introduced the *exponentiated generalized* (EG) family of distributions as an extension of exponentiated-type distributions, offering enhanced flexibility for modeling data in a wide range of scientific and engineering applications. For a non-negative continuous random variable X, the cdf of the EG family is defined by

(8) $$F\left(x;\theta ,\lambda \right)={\left[1-{\left(1-G(x)\right)}^{\theta }\right]}^{\lambda }\;,\;\theta ,\lambda >0,$$

where θ and λ are additional shape parameters. Consequently, the corresponding pdf is expressed as

(9) $$f\left(x;\theta ,\lambda \right)=\theta {\lambda \overset{´}{G}\left(x\right)\left(1-G\left(x\right)\right)}^{\theta -1}{\left[1-{\left(1-G(x)\right)}^{\theta }\right]}^{\lambda -1},\;\;\;\;\theta ,\lambda >0,$$

where$\;\overset{´}{G}\left(x\right)$ is the first derivative of G(x) with respect to x.

Since its introduction, the EG family has been extensively generalized and successfully applied to numerous baseline distributions. For instance, Ref. [34] proposed a generalization of the inverted exponential distribution, Ref. [35] introduced the transmuted EG–G family of distributions, Ref. [36] examined the EG-extended exponential distribution, Ref. [37] investigated the general exponentiated class of distributions, Ref. [38] developed the EG exponential Dagum distribution, Ref. [39] formulated the exponentiated exponential–exponentiated Weibull linear mixed distribution, Ref. [40] studied the EG power series family and its sub-models, Ref. [41] introduced the EG inverted Kumaraswamy distribution, Ref. [42] proposed another extension of the EG family, Ref. [43] developed the EG Weibull exponential distribution, and Ref. [44] introduced the EG xgamma distribution.

Combining the P distribution with the EG family yields a new and more flexible lifetime distribution, termed the *exponentiated generalized Pham* (EGP) distribution, capable of accommodating a wide variety of density and hazard rate shapes for modeling complex lifetime data. Although numerous extensions of the P distribution and applications of the EG family have been reported, existing studies have primarily focused on distributional properties and parameter estimation based on complete random samples. In contrast, entropy estimation under ordered sampling schemes has received little attention. In particular, no study has investigated the estimation of Shannon, Rényi, and Tsallis entropies for the EGP distribution within the unified frameworks of GOS and DGOS, which encompass ordinary OS, ROS, UR, and LR. This gap motivates the present paper.

Accordingly, this paper introduces the EGP distribution and develops a unified framework for entropy estimation under GOS and DGOS. The main contributions of this paper are summarized as follows:

1. Proposes the EGP distribution by combining the P distribution with the EG family, thereby providing a flexible lifetime model capable of accommodating a wide range of data behaviors.
2. Derives the fundamental statistical properties of the proposed distribution, including its quantile function, moments, skewness, kurtosis, mode, residual life, reversed residual life, and Shannon, Rényi, and Tsallis entropy measures.
3. Develops ML estimation procedures for the EGP model parameters and the associated entropy measures under the unified GOS and DGOS frameworks.
4. Evaluates the finite-sample performance of the proposed estimators through extensive simulation studies and demonstrates the practical applicability of the EGP distribution using real datasets, with comparisons against several competing lifetime models based on standard goodness-of-fit criteria.

The remainder of this article is organized as follows. Section 2 introduces the EGP distribution, detailing cdf, pdf, sf, and hrf. In addition, examining its statistical properties, including quantiles, moments, and graphical analyses of the pdf and hrf, illustrates the model’s shape flexibility, while entropy measures, Shannon, Rényi, and Tsallis, are derived analytically or numerically within the EGP framework. Section 3 addresses parameter and entropy estimation under GOS and DGOS, with results specialized to UR and LR values. Section 4 derives a numerical study, involving both a simulation study and two real datasets, evaluating estimation accuracy, model performance, and comparisons with competing distributions. Section 5 concludes with key theoretical and empirical findings and emphasizes the impact of GOS and DGOS on entropy.

## 2. Construction and Properties of the Exponentiated Generalized Pham Distribution

This section introduces the formulation of the EGP distribution and examines its probabilistic behavior through the shapes of the pdf and hrf. Furthermore, it presents the derivation of the key mathematical properties of the EGP distribution, which provides a deeper understanding of its structural flexibility and analytical tractability.

### 2.1. Formulation and Shape Characteristics of the EGP Distribution

Assume that X is a random variable distribution with EGP distribution with shape parameters α,θ,λ>0, and β>1, which can be obtained by substituting (6) into (8), denoted by *X* ~ EGP (α,β,θ,λ); the corresponding cdf and pdf are given, respectively, by:

(10) $$F\left(x;\alpha ,\beta ,\theta ,\lambda \right)={\left[1-e^{\theta \left(1-\beta^{x^{\alpha }}\right)}\right]}^{\lambda }\;,\alpha ,\theta ,\lambda >0,\beta >1,\;x>0,$$

and

(11) $$f\left(x;\alpha ,\beta ,\theta ,\lambda \right)=\theta \lambda \alpha \ln\left(\beta \right)x^{\alpha -1}\beta^{x^{\alpha }}e^{\theta \left(1-\beta^{x^{\alpha }}\right)}{\left[1-e^{\theta \left(1-\beta^{x^{\alpha }}\right)}\right]}^{\lambda -1},\;\;\;\;\;\;\;\;\;\;\;\;\;\;\;\;\;\;\;\;\;\;\;\;\;\;\;\;\;\;\;\;\;\;\;\;\;\;\;\;\;\;\;\;\;\;\;\;\;\;\;\;\;\;\;\;\;\;\;\;\;\;\;\alpha ,\theta ,\lambda >0,\;\;\;\;\;\beta >1,\;x>0.$$

The corresponding sf, hrf and *reversed hrf* (rhrf) are given, respectively, by

(12) $$S\left(x;\alpha ,\beta ,\theta ,\lambda \right)={1-\left[1-e^{\theta \left(1-\beta^{x^{\alpha }}\right)}\right]}^{\lambda }\;\;,\;\;\;\;\alpha ,\theta ,\lambda >0,\beta >1,\;x>0,$$

(13) $$h\left(x;\alpha ,\beta ,\theta ,\lambda \right)=\frac{\theta \lambda \alpha \ln\left(\beta \right)x^{\alpha -1}\beta^{x^{\alpha }}e^{\theta \left(1-\beta^{x^{\alpha }}\right)}{\left[1-e^{\theta \left(1-\beta^{x^{\alpha }}\right)}\right]}^{\lambda -1}}{{1-\left[1-e^{\theta \left(1-\beta^{x^{\alpha }}\right)}\right]}^{\lambda }},\;\alpha ,\theta ,\lambda >0,\beta >1,\;x>0,$$

and

(14) $$rh\left(x;\alpha ,\beta ,\theta ,\lambda \right)=\theta \lambda \alpha \ln\left(\beta \right)x^{\alpha -1}\beta^{x^{\alpha }}e^{\theta \left(1-\beta^{x^{\alpha }}\right)}{\left[1-e^{\theta \left(1-\beta^{x^{\alpha }}\right)}\right]}^{-1},\;\alpha ,\theta ,\lambda >0,\beta >1,\;x>0.\;$$

It is worth noting that the EGP distribution, as defined in (10) and (11), reduces to the extended P distribution proposed by [31] when θ=1, and further simplifies to the original P distribution proposed by [25] when θ=λ=1. These reductions follow directly from (10) and (11) by substituting the corresponding parameter values.

**Figure 1:**
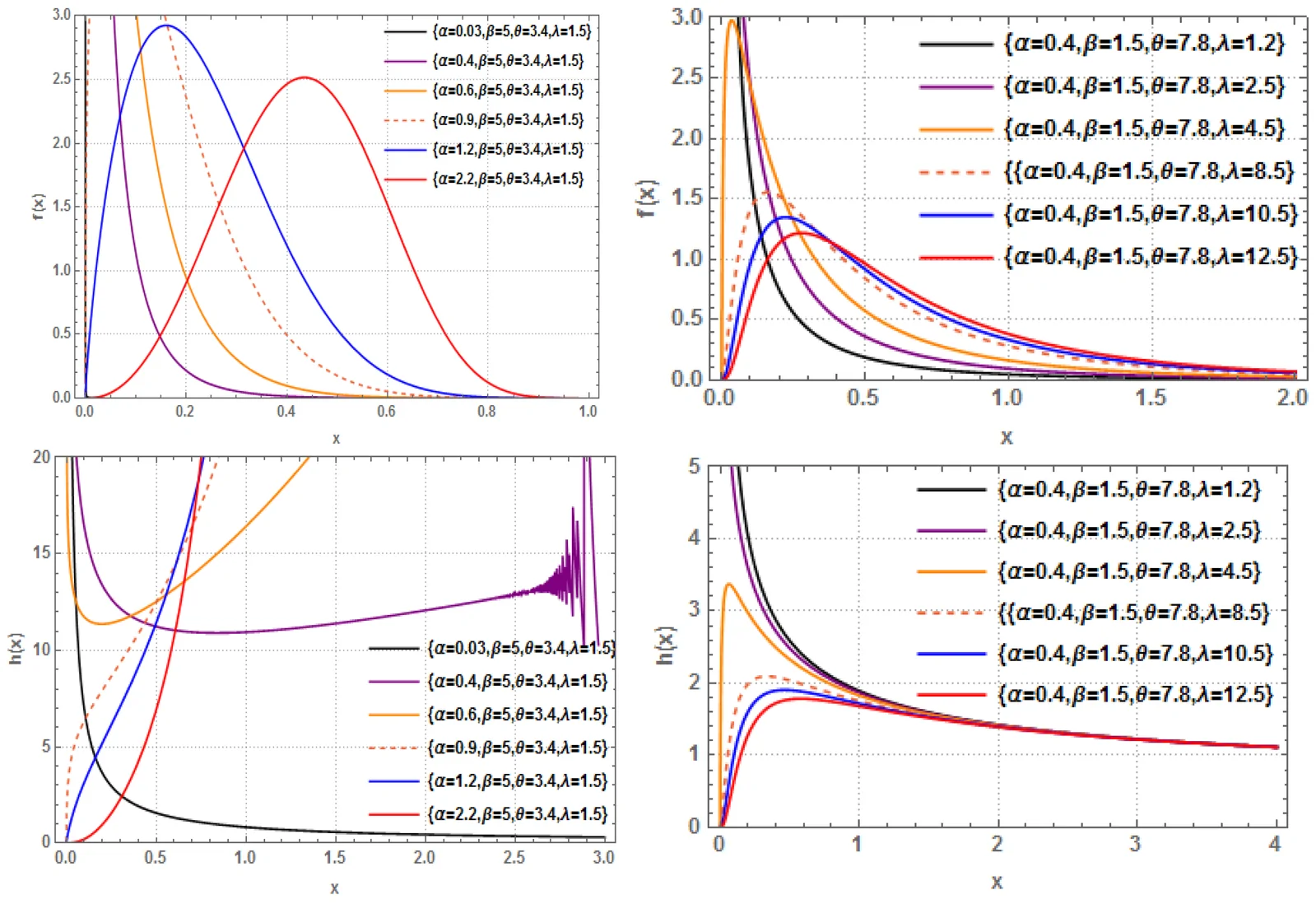
Plots of the pdf and hrf of the EGP distribution for various combinations of the parameters.

Figure 1 illustrates the diverse shapes of the pdf and hrf of the EGP distribution under various combinations of the model parameters. The figure clearly highlights the remarkable flexibility and practical relevance of the proposed model in representing a wide range of data behaviors. The pdf of the EGP distribution can exhibit several distinct forms, including J-shaped, semi-symmetric, and right-skewed patterns, depending on the parameter values, and is generally unimodal with a single peak. This adaptability enables the model to characterize both light- and heavy-tailed data structures commonly observed in survival, reliability, and lifetime analyses.

Similarly, the hrf of the EGP distribution can assume multiple forms, such as increasing, decreasing, non-monotonic decreasing, bathtub-shaped, or inverted Vtub-shaped patterns. These behaviors are of particular significance in reliability engineering, as they reflect different aging processes and failure mechanisms in systems or components. It is noteworthy that two specific shapes of the hrf, namely, the non-monotonic decreasing and the reversed Vtub-shaped forms are rarely documented in the existing literature. Nevertheless, these patterns hold considerable practical importance, as they correspond to realistic reliability behaviors encountered in various engineering contexts.

For example, certain electronic components may exhibit a non-monotonic decreasing hrf, operating efficiently for a period, and then undergoing temporary performance degradation followed by partial recovery. Likewise, a reversed Vtub-shaped hrf may arise in systems that initially experience a rapid increase in failure rate due to early stress or adaptation effects, followed by a subsequent decline as weaker units fail and more robust ones continue functioning. These examples underscore the capability of the EGP distribution to capture complex and practically relevant lifetime behaviors that traditional models often fail to represent adequately.

### 2.2. Mathematical Properties

The derivation of several statistical properties of the EGP distribution involves the use of the generalized integro-exponential function [45] and the incomplete generalized integro-exponential function [46]. These functions are defined, respectively, by the following integrals:

(15) $${\;E}_{c}^{d}\left(z\right)=\frac{1}{d!}\int_{1}^{\infty }{\left[\ln\left(u\right)\right]}^{d}u^{-c}e^{-zu}du,$$

and

(16) $${\;E}_{c}^{d}\left(z,h\right)=\frac{1}{d!}\int_{1}^{h}{\left[\ln\left(u\right)\right]}^{d}u^{-c}e^{-zu}du,$$

where c∈(−∞,∞),d>−1,z∈(−∞,∞) and $\underset{h\to \infty }{\lim}E_{c}^{d}\left(z,h\right)=E_{c}^{d}\left(z\right).$

- Quantiles

By inverting the cdf of the EGP distribution, given in (10), the quantile function is determined as follows:

(17) $$Q\left(b\right)={\left(\frac{\ln\left[1-\frac{\ln\left(1-b^{\frac{1}{\lambda }}\right)}{\theta }\right]}{\ln\left(\beta \right)}\right)}^{\frac{1}{\alpha }},\;0<b<1.$$

The median of the EGP distribution can be obtained by setting b= 0.5 in the above expression. Furthermore, by considering b as a uniform random variable on the interval (0, 1), random samples from the EGP distribution can be generated efficiently.

- Probability weighted moments

The *probability-weighted moments* (PWM), also known as power moments, are widely used in statistical inference and can be defined for a random variable X in terms of its cdf as

(18) $$M_{s,p,g}=E\left[X^{s}{\left(F\left(X\right)\right)}^{p}{\left(1-F\left(X\right)\right)}^{g}\right],\;$$

where p, g and s are any real numbers. Note that $\;M_{s,0,0}$ corresponds to the non-central moment of order s. For the EGP distribution, the PWM can be formulated by substituting (10) and (11) into (18) as

$$M_{s,p,g}=\theta \lambda \alpha \ln\left(\beta \right)\int_{0}^{\infty }x^{s+\alpha -1}\beta^{x^{\alpha }}e^{\theta \left(1-\beta^{x^{\alpha }}\right)}{\left[1-e^{\theta \left(1-\beta^{x^{\alpha }}\right)}\right]}^{\lambda \left(p+1\right)-1}\;\times {\left({1-\left[1-e^{\theta \left(1-\beta^{x^{\alpha }}\right)}\right]}^{\lambda }\right)}^{g}\;\;dx.$$

To facilitate analytical evaluation, one can employ the generalized binomial expansion:

(1−x)p=∑ι=0∞(−1)τ(pτ)xτ,  |x|≤1.

Applying this expansion twice to the integrand, the PWM can be rewritten as

Ms,p,g=θλαln(β)∑τ1=0∞∑τ2=0∞(gτ1)(λ(p+τ1+1)−1τ2)(−1)τ1+τ2eθ(τ2+1)                ×∫0∞xs+α−1βxαe−θ(τ2+1)βxα dx. 

Next, by making the substitution $\beta^{x^{\alpha }}=u,\;$the integral in the previous expression can be further simplified to be

Ms,p,g=θλ∑τ1=0∞∑τ2=0∞(gτ1)(λ(p+τ1+1)−1τ2)(−1)τ1+τ2(lnβ)−sαeθ(τ2+1)×∫1∞(lnu)sα e−θ(τ2+1)u  du.

By using the generalized integro-exponential function defined in (15), where $c\;\to \;0,\;d\;\to \;\frac{s}{\alpha }$, the PWM of the EGP distribution can be written as:

(19) Ms,p,g=θλ∑τ1=0∞∑τ2=0∞(gτ1)(λ(p+τ1+1)−1τ2)(−1)τ1+τ2[ln(β)]−sαeθ(τ2+1)×(sα!)E0sα[θ(τ2+1)].

When p=0, g=0, the $s^{th}$ non-central moment ${\;M}_{s,0,0}$ of the EGP distribution is derived to be

(20) E(Xs)=θλ∑τ2=0∞(−1)τ2(λ−1τ2)(lnβ)−sαeθ(τ2+1)(sα!)E0sα(θ(τ2+1)) .

Consequently, the *mean* (E(X)), *variance* (Var(X)), *skewness* (Sk(X)), *kurtosis* (Ku(X)) and *coefficient of variation* $\left(\gamma =\frac{\sqrt{Var(X)}}{E\left(X\right)}\right)\;$of the EGP distribution can be determined from (20). It follows from (20) that the raw moments depend on the parameter β only through the multiplicative factor ${\left(\ln\beta \right)}^{-\frac{s}{\alpha }}$. Since this factor is common to all raw moments, it cancels in the expressions for Sk(X), Ku(X), and γ. Consequently, these measures are independent of β.

Table 1 displays the numerical values of these measures for various combinations of the distribution parameters.

Table 1 demonstrates that variations in the parameters α, β, θ, and λ exert a systematic influence on the distribution’s key characteristics. The observed changes in the E(X) and Var(X) confirm the parameters’ direct effect on the distribution’s location and scale. Sk(X) exhibits fluctuations across different parameter combinations, highlighting the distribution’s capacity to capture both positive and negative asymmetry, a critical aspect in lifetime and reliability analyses where failure behaviors are often non-symmetric. Similarly, Ku(X) reflects the model’s flexibility in representing a wide range of tail behaviors, from light- to heavy-tailed patterns, thereby accommodating datasets with extreme values or outliers. Moreover, γ varies consistently with parameter adjustments, indicating precise control over relative dispersion. Together, these observations underscore the EGP distribution’s versatility and robustness, making it particularly well-suited for modeling complex lifetime and reliability data with diverse shapes, scales, and tail characteristics.

- Incomplete Moments

In lifetime and reliability studies, incomplete moments play a crucial role, particularly in the derivation of Lorenz and Bonferroni curves. For any continuous random variable X with pdf, f(x), the $s^{th}$ incomplete moment is defined as:

$$\varphi_{s}\left(t\right)=\int_{-\infty }^{t}x^{s}f\left(x\right)dx.\;$$

For the EGP distribution, using (11), the $s^{th}$ incomplete moment is expressed as:

$$\varphi_{s}\left(t\right)=\int_{-\infty }^{t}\theta \lambda \alpha \ln\left(\beta \right)x^{\alpha +s-1}\beta^{x^{\alpha }}e^{\theta \left(1-\beta^{x^{\alpha }}\right)}{\left[1-e^{\theta \left(1-\beta^{x^{\alpha }}\right)}\right]}^{\lambda -1}\;dx.$$

Following the same approach as in the derivation of the PWM,

φs(t)=θλαln(β)∑τ=0∞(λ−1τ)(−1)τeθ(τ+1)(sα!)[1(sα!)∫1βtα[ln(u)]sα e−θ(τ+1)u du].

Consider the incomplete generalized integro-exponential represented in (16), where $c\;\to \;0,\;d\;\to \;\frac{s}{\alpha }$, the $s^{th}$ incomplete moment of the EGP distribution can be represented as:

(21)                      φs(t)=θλαln(β)∑τ=0∞(λ−1τ)(−1)τeθ(τ+1)(sα!)E0(sα)(θ(τ+1),βtα) .. 

- Moments of residual life and reversed residual life functions

The residual life function represents a fundamental measure of aging and plays a central role in reliability theory and life-testing analyses. It provides valuable insight into the expected remaining lifetime of an item that has survived up to a given time t. The $s^{th}\;$moment of the residual life, denoted as $m_{s}\left(t\right)=E\left[{\left(X-t\right)}^{s}|X>t\right],s=1,2,\ldots$, is defined by:

(22) ms(t)=1S(x)∫t∞(x−t)sf(x)dx=1S(t)∑w=0s(sw)(−t)s−w∫t∞xwf(x)dx.

Accordingly, the $s^{th}$ moment of the residual life for the EGP distribution can be expressed in terms of the $w^{th}$ ordinary and incomplete moments as:

(23) ms(t)=1S(x;α,β,θ,λ)∑w=0s(sw)(−t)s−w[E(Xw)−φw(t)] , 

where S(x;α,β,θ,λ) denotes the sf of the EGP distribution as given in (12), while $E\left(X^{w}\right)$ and $\varphi_{w}\left(t\right)$ represent the $w^{th}$ complete and incomplete moments, respectively, which can be obtained by substituting s with w in (20) and (21). The mean residual life function, representing the expected remaining lifetime beyond time t, is obtained by setting s=1 in (23).

Analogously, the reversed residual life function, which describes the elapsed time since failure, is also of great significance in lifetime analysis. The$\;s^{th}$ moment of the reversed residual life is defined as:

(24) Φs(t)=1F(t)∑w=0s(sw)(t)s−w(−1)w∫0txwf(x)dx.

Using the definition of the incomplete moment given in (21), the $s^{th}$ moment of the reversed residual life for the EGP distribution can be expressed as:

(25) Φs(t)=1F(t)∑w=0s(sw)(t)s−w(−1)wφw(t). 

The mean inactivity time, also referred to as the mean waiting time, quantifies the expected time elapsed since failure, given that failure has occurred in the interval (0,t). For the EGP distribution, it can be obtained directly by setting s=1 in (25).

- Inequality measures

Incomplete moments, particularly the first incomplete moment, are central to the derivation of several widely used inequality measures, including the Lorenz, Bonferroni, and Zenga curves. While these curves were originally developed in economics to analyze income and wealth distributions, they have also found extensive applications in reliability analysis, demography, insurance, and medical research. These measures are formally defined as:

(26) $$L_{F}\left(t\right)=\frac{\int_{-\infty }^{t}xf\left(x\right)dx}{E\left(X\right)}=\frac{\varphi_{1}\left(t\right)}{E\left(X\right)}\;,\;$$

(27) $$B_{F}\left(t\right)=\frac{\int_{-\infty }^{t}xf\left(x\right)dx}{E\left(X\right)F(t)}=\frac{L_{F}\left(t\right)}{F(t)},$$

and

(28) $$A_{F}\left(t\right)=1-\frac{M^{-}\left(t\right)}{M^{+}\left(t\right)}\;,\;$$

where $M^{-}\left(t\right)=\frac{\int_{-\infty }^{t}xf\left(x\right)dx}{F\left(t\right)}=\;\frac{\varphi_{1}\left(t\right)}{F\left(t\right)}$, and $M^{+}\left(t\right)=\frac{\int_{t}^{\infty }xf\left(x\right)dx}{1-F\left(t\right)}=\;\frac{E\left(X\right)-\varphi_{1}\left(t\right)}{1-F\left(t\right)}$.

These functions can be evaluated precisely for the EGP distribution using (26)–(28), thereby providing a rigorous framework for quantifying inequality or variability in both economic and reliability contexts.

**Figure 2:**
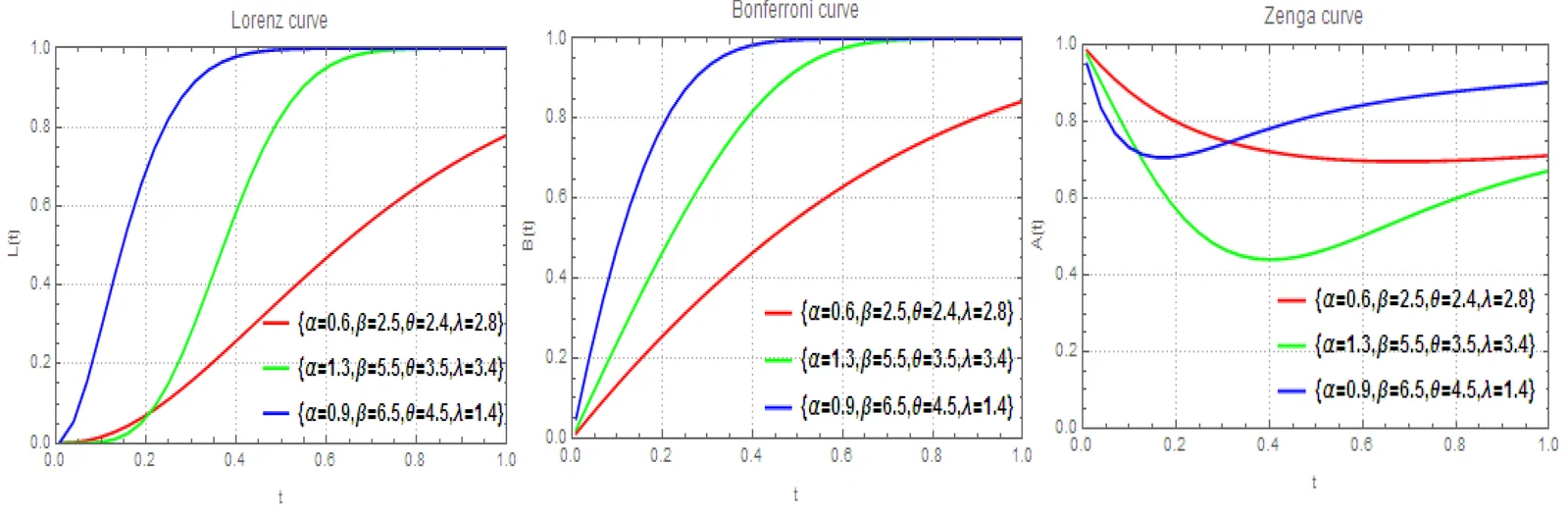
Lorenz, Bonferroni, and Zenga curves of the EGP distribution for selected parameter values.

Figure 2 presents the Lorenz, Bonferroni, and Zenga curves of the EGP distribution for different parameter combinations. These curves demonstrate the flexibility of the proposed distribution in capturing low, moderate, and high levels of inequality through appropriate choices of the model parameters. As the parameter values vary, noticeable changes in the curvature of the three curves are observed, reflecting different degrees of concentration and dispersion. This behavior highlights the ability of the EGP distribution to model data exhibiting diverse patterns of heterogeneity and inequality.

- Entropies of EGP distribution

A key objective of this study is to investigate and quantify the entropy measures associated with the EGP distribution. Analyzing these entropy measures offers meaningful insight into the model’s ability to capture different degrees of randomness, uncertainty, and heterogeneity in data. By applying the definitions of entropies given in (1), (2), and (3), together with the pdf of the EGP distribution presented in (11), the Shannon, Rényi, and Tsallis entropy measures can be derived. The Shannon entropy of a random variable X follows the EGP distribution defined as:

(29) $$HS\left(f\right)=-\int_{0}^{\infty }\theta \lambda \alpha \ln\left(\beta \right)x^{\alpha -1}\beta^{x^{\alpha }}e^{\theta \left(1-\beta^{x^{\alpha }}\right)}{\left[1-e^{\theta \left(1-\beta^{x^{\alpha }}\right)}\right]}^{\lambda -1}\;\;\;\;\;\;\;\;\;\;\;\;\;\;\;\;\;\;\;\;\;\;\;\times \ln\left[\theta \lambda \alpha \ln\left(\beta \right)x^{\alpha -1}\beta^{x^{\alpha }}e^{\theta \left(1-\beta^{x^{\alpha }}\right)}{\left[1-e^{\theta \left(1-\beta^{x^{\alpha }}\right)}\right]}^{\lambda -1}\right]dx.$$

Substituting (11) into (2) yields the specific form of the Rényi entropy for the EGP distribution. Further, the Rényi entropy of a random variable X that follows EGP is expressed as

(30) $$HR_{p}\left(f\right)=\frac{1}{1-p}\ln\left[\int_{-\infty }^{\infty }{\left[\theta \lambda \alpha \ln\left(\beta \right)x^{\alpha -1}\beta^{x^{\alpha }}e^{\theta \left(1-\beta^{x^{\alpha }}\right)}{\left[1-e^{\theta \left(1-\beta^{x^{\alpha }}\right)}\right]}^{\lambda -1}\right]}^{p}dx\right],\;\;p>0,p\neq 1.$$

Let $I={\left[\theta \lambda \alpha \ln\left(\beta \right)\right]}^{p}\int_{0}^{\infty }x^{p\left(\alpha -1\right)}{\left[\beta^{x^{\alpha }}\right]}^{p}e^{p\theta \left(1-\beta^{x^{\alpha }}\right)}{\left[1-e^{\theta \left(1-\beta^{x^{\alpha }}\right)}\right]}^{p\left(\lambda -1\right)}dx$; by using the generalized binomial expansion:

(1−x)p=∑τ=0∞(−1)τ(pτ)xτ, |x|≤1.

Therefore, I=[θλαln(β)]p∑τ=0∞(−1)τ(p(λ−1)τ)eθ(p+τ)∫0∞xp(α−1)[βxα]pe−θβxα(p+τ)dx.

Next, by making the substitution $\beta^{x^{\alpha }}=u,\;$the integral in the previous expression can be further simplified to be

I=(θλ)pαp−1[ln(β)]pα−1α+1∑τ=0∞(−1)τ(p(λ−1)τ)eθ(p+τ)∫1∞(lnu)p(1−1α)+1α−1up e−θ(p+τ)u du.

Consequently, the integral I can be expressed in terms of the generalized integro-exponential function defined in (15), where $c\;\to -p,\;d\;\to \;p\left(1-\frac{1}{\alpha }\right)+\frac{1}{\alpha }-1,\;$as

I=(θλ)pαp−1[ln(β)]pα−1α+1∑τ=0∞(−1)τ(p(λ−1)τ)eθ(p+τ)                                                ×[(p(1−1α)+1α−1)!]E(−p)p(1−1α)+1α−1[θ(p+τ)],

Then,

(31) HRp(f)=11−pln[ (θλ)pαp−1[ln(β)]pα−1α+1∑τ=0∞(−1)τ(p(λ−1)τ)eθ(p+τ)×[(p(1−1α)+1α−1)!]E(−p)p(1−1α)+1α−1(θ(p+τ))], 

while the Tsallis entropy of order q is defined as

(32) HTq(f)=1q−1{ 1−[(θλ)qαq−1[ln(β)]qα−1α+1∑τ=0∞(−1)τ(q(λ−1)τ)eθ(q+τ)                                                ×[(q(1−1α)+1α−1)!]E(−q)q(1−1α)+1α−1(θ(q+τ))]}.

These entropy measures provide a comprehensive characterization of the uncertainty associated with the EGP distribution. For continuous random variables, Shannon entropy (also referred to as differential entropy) quantifies the average uncertainty or information content of a distribution. Unlike its discrete analog, differential entropy does not possess an absolute scale and may assume negative values depending on the measurement units and the underlying distribution parameters. Moreover, its numerical evaluation requires integration, which may fail to converge even when the corresponding theoretical integral exists [1,47]. The numerical values of the Shannon, Rényi, and Tsallis entropy measures for the EGP distribution under various parameter combinations are presented in Table 2. These values were obtained by direct numerical evaluation of the defining entropy integrals using *Mathematica* 11. For parameter combinations in which the numerical integration algorithm did not converge, the corresponding entries are reported as *does not converge* (DC), indicating that a reliable numerical approximation could not be obtained using the adopted numerical integration procedure.

## 3. Estimation Methodology

This section presents a rigorous framework for estimating the parameters of the EGP distribution and the corresponding entropy measures. The methodology is designed to accommodate a range of sampling schemes, including GOS, DGOS, and special cases such as OS and ROS, as well as UR and LR values. Parameter estimation is conducted via the ML estimation approach. Approximate *confidence intervals* (CIs) for the model parameters are constructed using the observed Fisher information matrix, whereas those for the entropy measures are obtained via the Delta method. This unified framework ensures coherent inference across complete, censored, and ordered datasets.

### 3.1. Estimation Under GOS

Suppose that $X_{\left(1,n,m,k\right)},\;X_{\left(2,n,m,k\right)},\;{\ldots \;X}_{\left(n,n,m,k\right)}\;$denote a GOS sample of size n drawn from the EGP distribution with parameter vector Θ=(α, β, θ, λ), where α, θ, λ>0 and β>1. According to [12], the likelihood function for a GOS sample can be formulated by substituting (10) and (11) into (4). Thus, the likelihood function can be expressed as

(33) $$L\left(\Theta ;\underline{x}\right)=C\left[\prod_{i=1}^{n-1}{\left(1-{\left[1-e^{\theta \left(1-\beta^{{\left(x_{i}\right)}^{\alpha }}\right)}\right]}^{\lambda }\right)}^{m}\right]{\;\left(1-{\left[1-e^{\theta \left(1-\beta^{{\left(x_{n}\right)}^{\alpha }}\right)}\right]}^{\lambda }\right)}^{k-1}\;\;\times \left[\prod_{i=1}^{n}\theta \lambda \alpha \ln\left(\beta \right){\left(x_{i}\right)}^{\alpha -1}\beta^{{\left(x_{i}\right)}^{\alpha }}e^{\theta \left(1-\beta^{{\left(x_{i}\right)}^{\alpha }}\right)}{\left[1-e^{\theta \left(1-\beta^{{\left(x_{i}\right)}^{\alpha }}\right)}\right]}^{\lambda -1}\right],$$

where C is a normalizing constant depending on the GOS design parameters$,\;\underline{x}=x_{1},x_{2},\ldots ,x_{n},\;m,\;and\;k$. Accordingly, the log-likelihood function is given by

(34) $$l\propto n\;ln\theta +n\;ln\lambda +n\;ln\alpha +nln\left[\ln\left(\beta )\right]+\left(\alpha -1\right)\sum_{i=1}^{n}{ln[x}_{i}\right]+\sum_{i=1}^{n}ln\left[\beta^{x_{i}^{\alpha }}\right]+\theta \sum_{i-1}^{n}\left(1-\beta^{x_{i}^{\alpha }}\right)\;+\left[\lambda -1\right]\sum_{i=1}^{n}\ln\left[1-e^{\theta \left(1-\beta^{x_{i}^{\alpha }}\right)}\right]+\sum_{i=1}^{n-1}m\ln\left[1-{\left[1-e^{\theta \left(1-\beta^{{\left(x_{i}\right)}^{\alpha }}\right)}\right]}^{\lambda }\right]\;+\left(k-1\right)\ln\left[1-{\left[1-e^{\theta \left(1-\beta^{{\left(x_{n}\right)}^{\alpha }}\right)}\right]}^{\lambda }\right].$$

Differentiating l with respect to each parameter and setting the derivatives to zero yields the score equations:

(35) $$\frac{\partial l}{\partial \alpha }=\;\frac{n}{\alpha }+\sum_{i=1}^{n}{ln[x}_{i}]+\sum_{i=1}^{n}\frac{{x_{i}^{\alpha }\ln{[x}_{i}]\beta }^{x_{i}^{\alpha }}\ln\left[\beta \right]}{\beta^{x_{i}^{\alpha }}}-\theta \sum_{i-1}^{n}\frac{{x_{i}^{\alpha }\ln\left[x_{i}\right]\beta }^{x_{i}^{\alpha }}\ln\left[\beta \right]}{\left(1-\beta^{x_{i}^{\alpha }}\right)}\;+\left[\lambda -1\right]\sum_{i=1}^{n}\frac{{{\theta x}_{i}^{\alpha }\ln[x_{i}]\beta }^{x_{i}^{\alpha }}\ln\left[\beta \right]e^{\theta \left(1-\beta^{x_{i}^{\alpha }}\right)}}{\left[1-e^{\theta \left(1-\beta^{x_{i}^{\alpha }}\right)}\right]}\;-\sum_{i=1}^{n-1}\frac{m\left(\lambda \theta \ln\left[\beta \right]\right){x_{i}^{\alpha }\ln[x_{i}]\beta }^{x_{i}^{\alpha }}e^{\theta \left(1-\beta^{x_{i}^{\alpha }}\right)}{\left[1-e^{\theta \left(1-\beta^{{\left(x_{i}\right)}^{\alpha }}\right)}\right]}^{\lambda -1}}{\left[1-{\left[1-e^{\theta \left(1-\beta^{{\left(x_{i}\right)}^{\alpha }}\right)}\right]}^{\lambda }\right]}\;-\frac{{{\left(k-1\right)\lambda \theta \ln\left[\beta \right]x}_{n}^{\alpha }\ln[x_{n}]}^{x_{n}^{\alpha }}e^{\theta \left(1-\beta^{x_{n}^{\alpha }}\right)}{\left[1-e^{\theta \left(1-\beta^{{\left(x_{n}\right)}^{\alpha }}\right)}\right]}^{\lambda -1}}{\left[1-{\left[1-e^{\theta \left(1-\beta^{{\left(x_{n}\right)}^{\alpha }}\right)}\right]}^{\lambda }\right]},$$

(36) $$\frac{\partial l}{\partial \beta }=\frac{n}{\ln\left[\beta \right]}+\sum_{i=1}^{n}\frac{x_{i}\beta^{x_{i}^{\alpha }-1}}{\left[\beta^{x_{i}^{\alpha }}\right]}-\theta \sum_{i-1}^{n}\left(1-\beta^{x_{i}^{\alpha }}\right)\;x_{i}^{\alpha }\beta^{x_{i}^{\alpha }-1}+\left[\lambda -1\right]\sum_{i=1}^{n}\frac{\theta x_{i}^{\alpha }\beta^{x_{i}^{\alpha }-1}e^{\theta \left(1-\beta^{x_{i}^{\alpha }}\right)}}{\left[1-e^{\theta \left(1-\beta^{x_{i}^{\alpha }}\right)}\right]}\;-m\sum_{i=1}^{n-1}\frac{{\lambda \theta x_{i}^{\alpha }\beta^{x_{i}^{\alpha }-1}e^{\theta \left(1-\beta^{x_{i}^{\alpha }}\right)}\left[1-e^{\theta \left(1-\beta^{{\left(x_{i}\right)}^{\alpha }}\right)}\right]}^{\lambda -1}}{\left[1-{\left[1-e^{\theta \left(1-\beta^{{\left(x_{i}\right)}^{\alpha }}\right)}\right]}^{\lambda }\right]}\;+\frac{\left(k-1\right){\lambda \theta x_{n}^{\alpha }\beta^{x_{n}^{\alpha }-1}e^{\theta \left(1-\beta^{x_{n}^{\alpha }}\right)}\left[1-e^{\theta \left(1-\beta^{{\left(x_{n}\right)}^{\alpha }}\right)}\right]}^{\lambda -1}}{\left[1-{\left[1-e^{\theta \left(1-\beta^{{\left(x_{n}\right)}^{\alpha }}\right)}\right]}^{\lambda }\right]},$$

(37) $$\frac{\partial l}{\partial \theta }=\frac{n}{\theta }+\sum_{i-1}^{n}\left(1-\beta^{x_{i}^{\alpha }}\right)-\left[\lambda -1\right]\sum_{i=1}^{n}\frac{\left(1-\beta^{x_{i}^{\alpha }}\right)e^{\theta \left(1-\beta^{x_{i}^{\alpha }}\right)}}{\left[1-e^{\theta \left(1-\beta^{x_{i}^{\alpha }}\right)}\right]}\;+m\sum_{i=1}^{n-1}\frac{{\lambda \left(1-\beta^{x_{i}^{\alpha }}\right)e^{\theta \left(1-\beta^{x_{i}^{\alpha }}\right)}\left[1-e^{\theta \left(1-\beta^{{\left(x_{i}\right)}^{\alpha }}\right)}\right]}^{\lambda -1}}{\left[1-{\left[1-e^{\theta \left(1-\beta^{{\left(x_{i}\right)}^{\alpha }}\right)}\right]}^{\lambda }\right]}\;+\frac{\left(k-1\right){\lambda \left(1-\beta^{x_{n}^{\alpha }}\right)e^{\theta \left(1-\beta^{x_{n}^{\alpha }}\right)}\left[1-e^{\theta \left(1-\beta^{{\left(x_{n}\right)}^{\alpha }}\right)}\right]}^{\lambda -1}}{\left[1-{\left[1-e^{\theta \left(1-\beta^{{\left(x_{n}\right)}^{\alpha }}\right)}\right]}^{\lambda }\right]},$$

and

(38) $$\frac{\partial l}{\partial \lambda }=\frac{n}{\lambda }+\sum_{i=1}^{n}\ln\left[1-e^{\theta \left(1-\beta^{x_{i}^{\alpha }}\right)}\right]\;+m\sum_{i=1}^{n-1}\frac{{\left[1-e^{\theta \left(1-\beta^{{\left(x_{i}\right)}^{\alpha }}\right)}\right]}^{\lambda }\ln\left[1-e^{\theta \left(1-\beta^{{\left(x_{i}\right)}^{\alpha }}\right)}\right]}{\left[1-{\left[1-e^{\theta \left(1-\beta^{{\left(x_{i}\right)}^{\alpha }}\right)}\right]}^{\lambda }\right]}\;-\frac{\left(k-1\right){\left[1-e^{\theta \left(1-\beta^{{\left(x_{n}\right)}^{\alpha }}\right)}\right]}^{\lambda }\ln\left[1-e^{\theta \left(1-\beta^{{\left(x_{n}\right)}^{\alpha }}\right)}\right]}{\left[1-{\left[1-e^{\theta \left(1-\beta^{{\left(x_{n}\right)}^{\alpha }}\right)}\right]}^{\lambda }\right]}.$$

Since (35)–(38) are nonlinear and analytically intractable, closed-form solutions are not available. Therefore, the ML estimators based on GOS, denoted by $\left(\hat{\alpha },\;\hat{\beta },\;\hat{\theta },\;\hat{\lambda }\right)$, are obtained numerically using iterative procedures such as the Newton–Raphson method or other gradient-based optimization algorithms.

Under standard regularity conditions, the ML estimators of the model parameters are consistent, asymptotically efficient, and asymptotically normally distributed. The asymptotic covariance matrix of the estimators can be approximated by the inverse of the observed Fisher information matrix. The observed Fisher information matrix is given by

$$I_{O}\left(\Theta \right)={-\frac{\partial^{2}l}{\partial \Theta_{i}\partial \Theta_{j}}|}_{\Theta =\hat{\Theta }},\;i,j=1,\ldots ,\;4,$$

where $\hat{\Theta }=\left(\hat{\alpha },\hat{\beta },\hat{\theta },\hat{\lambda }\right)\;$denotes the vector of ML estimators under the GOS scheme. Accordingly, the estimated asymptotic covariance matrix is approximated by$\;I_{O}^{-1}\left(\Theta \right)$. Consequently, approximate 100(1 − ω) % CIs for the parameters can be constructed as follows:

(39) $$\;\;\hat{\alpha }\pm \;{\;Z}_{\left(1-\frac{\omega }{2}\right)}\sqrt{\tilde{var}\left(\hat{\alpha }\right)},\;\;\;\;\;\;\;\;\;\;\;\;\;\;\;\;\;\;\;\;\;\;\;\;\;\;\;\;\hat{\beta }\pm \;{\;Z}_{\left(1-\frac{\omega }{2}\right)}\sqrt{\tilde{var}\left(\hat{\beta }\right)}\;,\;$$

and

(40) $$\;\;\;\;\hat{\lambda }\pm \;{\;Z}_{\left(1-\frac{\omega }{2}\right)}\sqrt{\tilde{var}\left(\hat{\lambda }\right)},\;\;\;\;\;\;\;\;\;\;\;\;\;\;\;\;\;\;\;\;\;\;\;and\;\;\hat{\theta }\pm \;{\;Z}_{\left(1-\frac{\omega }{2}\right)}\sqrt{\tilde{var}\left(\hat{\theta }\right)}\;,\;$$

where ${\;Z}_{\left(1-\frac{\omega }{2}\right)}\;$denotes the $\left(1-\frac{\omega }{2}\right)$ quantile of the standard normal distribution and $\tilde{var}\left(\hat{.}\right)\;$represents the estimated asymptotic variance of the corresponding parameter obtained from the diagonal elements of $I_{O}^{-1}\left(\Theta \right)$. It is noteworthy that the ML estimation of the distribution parameters under the GOS framework can be reduced to several important special cases. Specifically, when m=0 and k=1. The ML estimators correspond to those obtained under the OS model. Moreover, setting m=−1 and k=1 yields the ML estimators based on the UR values.

### 3.2. Estimation Under DGOS

The DGOS model represents the reverse counterpart of the GOS framework, where descending sequences of ordered random variables are considered, thereby offering increased flexibility in modeling diverse censoring and ranking schemes. This subsection develops the estimation procedure for the parameters of the EGP distribution when the data follow the DGOS model.

Let $T_{\left(1,n,m,k\right)},\;T_{\left(2,n,m,k\right)},\;{\ldots \;T}_{\left(n,n,m,k\right)}\;$denote a DGOS sample of size *n* drawn from the EGP distribution. Substituting the EGP distribution and density functions given in (10) and (11) into the general DGOS likelihood expression (5) yields the following likelihood function:

(41) $$L^{*}\left(\Theta ;\underline{t}\right)=C^{*}\theta^{n}\lambda^{n}\alpha^{n}{\left[\ln\left(\beta \right)\right]}^{n}\prod_{i=1}^{n}t_{i}^{\alpha -1}\beta^{t_{i}^{\alpha }}e^{\theta \left(1-\beta^{t_{i}^{\alpha }}\right)}{\left[1-e^{\theta \left(1-\beta^{t_{n}^{\alpha }}\right)}\right]}^{\lambda k-1}\;\;\times \left[\prod_{i=1}^{n-1}{\left[1-e^{\theta \left(1-\beta^{t_{i}^{\alpha }}\right)}\right]}^{\lambda \left(m+1\right)-1}\right],\;$$

where$\;C^{*}\;$is a normalizing constant depending on the DGOS design parameters, $\underline{t}=t_{1},t_{2},\ldots ,t_{n},\;m,\;and\;k.$ Accordingly, taking the natural logarithm of (41) leads to the log-likelihood function:

(42) $$l^{*}=n\;ln\theta +n\;ln\lambda +n\;ln\alpha +n\;ln[\ln\left(\beta )\right]+\left(\lambda k-1\right)\ln\left[1-e^{\theta \left(1-\beta^{t_{n}^{\alpha }}\right)}\right]+(\alpha -1)\sum_{i=1}^{n}t_{i}\;+\sum_{i=1}^{n}ln\left(\beta^{t_{i}^{\alpha }}\right)+\sum_{i-1}^{n}\theta \left(1-\beta^{t_{i}^{\alpha }}\right)+\left[\lambda \left(m+1\right)-1\right]\sum_{i=1}^{n-1}\ln\left[1-e^{\theta \left(1-\beta^{t_{i}^{\alpha }}\right)}\right].\;$$

To obtain the ML estimators of the parameters α,β,θ and λ, one differentiates (42) with respect to each parameter and equates the resulting expressions to zero. The corresponding score equations are given by:

(43) $$\frac{\partial l^{*}}{\partial \alpha }=\frac{n}{\alpha }+\sum_{i=1}^{n}t_{i}+\sum_{i=1}^{n}\frac{{t_{i}^{\alpha }\ln t_{i}\beta }^{t_{i}^{\alpha }}\ln\beta }{\beta^{t_{i}^{\alpha }}}\;+\left[\lambda \left(m+1\right)-1\right]\sum_{i=1}^{n-1}\frac{e^{\theta \left(1-\beta^{t_{i}^{\alpha }}\right)}\theta {t_{i}^{\alpha }\ln t_{i}\beta }^{t_{i}^{\alpha }}\ln\beta }{\left[1-e^{\theta \left(1-\beta^{t_{i}^{\alpha }}\right)}\right]}\;+\theta \sum_{i-1}^{n}{t_{i}^{\alpha }\ln t_{i}\beta }^{t_{i}^{\alpha }}\ln\beta +\left(\lambda k-1\right)\frac{e^{\theta \left(1-\beta^{t_{n}^{\alpha }}\right)}\theta {t_{n}^{\alpha }\ln t_{n}\beta }^{t_{n}^{\alpha }}\ln\beta }{\left[1-e^{\theta \left(1-\beta^{t_{n}^{\alpha }}\right)}\right]}\;,\;\;$$

(44) $$\frac{\partial l^{*}}{\partial \beta }=\frac{n}{\ln\left(\beta \right)}+\sum_{i=1}^{n}\frac{{t_{i}^{\alpha }\beta }^{t_{i}^{\alpha }-1}}{\beta^{t_{i}^{\alpha }}}+\theta \sum_{i-1}^{n}{t_{i}^{\alpha }\beta }^{t_{i}^{\alpha }-1}+\left[\lambda \left(m+1\right)-1\right]\sum_{i=1}^{n-1}\frac{{{{\theta t}_{i}^{\alpha }\beta }^{t_{i}^{\alpha }-1}e}^{\theta \left(1-\beta^{t_{i}^{\alpha }}\right)}}{\left[1-e^{\theta \left(1-\beta^{t_{i}^{\alpha }}\right)}\right]}\;+\left(\lambda k-1\right)\frac{{{{\theta t}_{n}^{\alpha }\beta }^{t_{n}^{\alpha }-1}e}^{\theta \left(1-\beta^{t_{n}^{\alpha }}\right)}}{\left[1-e^{\theta \left(1-\beta^{t_{n}^{\alpha }}\right)}\right]},$$

(45) $$\frac{\partial l^{*}}{\partial \theta }=-\left[\lambda \left(m+1\right)-1\right]\sum_{i=1}^{n-1}\left\{\frac{\left(1-\beta^{t_{i}^{\alpha }}\right)e^{\theta \left(1-\beta^{t_{i}^{\alpha }}\right)}}{\left[1-e^{\theta \left(1-\beta^{t_{i}^{\alpha }}\right)}\right]}-\left(\lambda k-1\right)\frac{\left(1-\beta^{t_{i}^{\alpha }}\right)e^{\theta \left(1-\beta^{t_{n}^{\alpha }}\right)}}{\left[1-e^{\theta \left(1-\beta^{t_{n}^{\alpha }}\right)}\right]}\right\}\;\;+\frac{n}{\theta }+\sum_{i-1}^{n}\left(1-\beta^{t_{i}^{\alpha }}\right),$$

and

(46) $$\frac{\partial l^{*}}{\partial \lambda }=\frac{n}{\lambda }+\left(m+1\right)\sum_{i=1}^{n-1}\ln\left[1-e^{\theta \left(1-\beta^{t_{i}^{\alpha }}\right)}\right]+k\ln\left[1-e^{\theta \left(1-\beta^{t_{n}^{\alpha }}\right)}\right].$$

Since these equations are nonlinear and mutually dependent, closed-form analytical solutions are generally infeasible. Therefore, numerical optimization methods, such as the Newton–Raphson algorithm or other iterative procedures, are utilized to obtain the estimates $\hat{\alpha },\hat{\beta },\hat{\theta }\;and\;\hat{\lambda }$. However, it is noteworthy that the parameter λ permits an explicit expression by setting (46) to zero, from which a direct estimate can be derived.

(47) $$\hat{\lambda }=\frac{-n}{\left(m+1\right)\sum_{i=1}^{n-1}\ln\left[1-e^{\hat{\theta }\left(1-{\hat{\beta }}^{t_{i}^{\hat{\alpha }}}\right)}\right]+k\ln\left[1-e^{\hat{\theta }\left(1-{\hat{\beta }}^{t_{n}^{\hat{\alpha }}}\right)}\right]\;}.$$

Under standard regularity conditions, the ML estimators are consistent, asymptotically unbiased, and normally distributed. The asymptotic covariance matrix of the estimators is approximated by the inverse of the observed Fisher information matrix. The observed Fisher information matrix under DGOS schema is defined as

$$I_{0}^{*}\left(\Theta \right)={-\frac{\partial^{2}l^{*}}{\partial \Theta_{i}\partial \Theta_{j}}|}_{\Theta =\hat{\Theta }},\;i,j=1,\ldots ,\;4,$$

where $\hat{\Theta }=\left(\hat{\alpha },\hat{\beta },\hat{\theta },\hat{\lambda }\right)$ denotes the vector of ML estimators under the DGOS scheme. Consequently, the estimated asymptotic covariance matrix is approximated by ${\left(I_{0}^{*}\left(\Theta \right)\right)}^{-1}$. Hence, approximate 100(1 −  ω)% CIs for each parameter can be constructed as

(48) $$\hat{\alpha }\pm \;{\;Z}_{\left(1-\frac{\omega }{2}\right)}\sqrt{\tilde{var}\left(\hat{\alpha }\right)},\;\;\;\;\;\;\;\;\;\;\;\;\;\hat{\beta }\pm \;{\;Z}_{\left(1-\frac{\omega }{2}\right)}\sqrt{\tilde{var}\left(\hat{\beta }\right)}\;,$$

(49) $$\hat{\lambda }\pm \;{\;Z}_{\left(1-\frac{\omega }{2}\right)}\sqrt{\tilde{var}\left(\hat{\lambda }\right)},\;\;\;\;\;\;\;\;\;\;\;\;\;\;\;\;\;\;and\;\;\hat{\theta }\pm \;{\;Z}_{\left(1-\frac{\omega }{2}\right)}\sqrt{\tilde{var}\left(\hat{\theta }\right)}\;,\;$$

where ${\;Z}_{\left(1-\frac{\omega }{2}\right)}\;$ is the quantile of the standard normal distribution and $\tilde{var}\left(\hat{.}\right)$ denotes the estimated asymptotic variance obtained from the diagonal elements of ${\left(I_{0}^{*}\left(\Theta \right)\right)}^{-1}$.

Importantly, the DGOS framework encompasses several special cases. Setting m=−1 and k=1 reduces the model to LR values, whereas setting m=0 and k=1 corresponds to ROS. Therefore, the ML estimators of the EGP distribution can be obtained directly from these sampling schemes.

### 3.3. Entropy Estimation

Entropy measures provide a formal quantification of the uncertainty and information content inherent in a pdf. For the EGP distribution, we focus on Shannon, Rényi, and Tsallis entropies. By virtue of the invariance property of ML estimation, any function of the model parameters can be estimated by substituting the ML estimators into its theoretical expression. Consequently, substituting the estimators obtained under either the GOS or DGOS frameworks into (29), (31), and (32) yields the corresponding estimators of the entropies as [1], given by

(50) $$\tilde{HS}\left(f\right)=-\int_{0}^{\infty }\theta_{es}\lambda_{es}\alpha_{es}\ln\left(\beta_{es}\right)x^{\alpha_{es}-1}{\beta_{es}}^{x^{\alpha_{es}}}e^{\theta_{es}\left(1-{\beta_{es}}^{x^{\alpha_{es}}}\right)}{\left[1-e^{\theta_{es}\left(1-{\beta_{es}}^{x^{\alpha_{es}}}\right)}\right]}^{\lambda_{es}-1}\;\times \ln\left[\theta_{es}\lambda_{es}\alpha_{es}\ln\left(\beta_{es}\right)x^{\alpha_{es}-1}{\beta_{es}}^{x^{\alpha_{es}}}e^{\theta_{es}\left(1-{\beta_{es}}^{x^{\alpha_{es}}}\right)}{\left[1-e^{\theta_{es}\left(1-{\beta_{es}}^{x^{\alpha_{es}}}\right)}\right]}^{\lambda_{es}-1}\right]dx,$$

and the Rényi entropy is

(51) $${\tilde{HR}}_{p}\left(f\right)=\frac{1}{1-p}\ln[\int_{0}^{\infty }{\left[\theta_{es}\lambda_{es}\alpha_{es}\ln\left(\beta_{es}\right)\right]}^{p}x^{p\left(\alpha_{es}-1\right)}{\beta_{es}}^{p\;x^{\alpha_{es}}}e^{p\;\theta_{es}\left(1-{\beta_{es}}^{x^{\alpha_{es}}}\right)}\;\times {\left[1-e^{\theta_{es}\left(1-{\beta_{es}}^{x^{\alpha_{es}}}\right)}\right]}^{p\left(\lambda_{es}-1\right)}dx].$$

Additionally, the Tsallis entropy is

(52) $${\tilde{HT}}_{q}\left(f\right)=\frac{1}{q-1}\{1-\int_{0}^{\infty }{\left[\theta_{es}\lambda_{es}\alpha_{es}\ln\left(\beta_{es}\right)\right]}^{q}x^{q\left(\alpha_{es}-1\right)}{\beta_{es}}^{q\;x^{\alpha_{es}}}\;\times e^{{q\;\theta }_{es}\left(1-{\beta_{es}}^{x^{\alpha_{es}}}\right)}{\left[1-e^{\theta_{es}\left(1-{\beta_{es}}^{x^{\alpha_{es}}}\right)}\right]}^{q\;\left(\lambda_{es}-1\right)}dx\},\;$$

where $\Theta_{es}\;$denotes the estimated parameter vector obtained under either the GOS $\left(\hat{\alpha },\;\hat{\beta },\;\hat{\theta },\;\hat{\lambda }\right)$ or DGOS $\left(\hat{\alpha },\;\hat{\beta },\;\hat{\theta },\;\hat{\lambda }\right)$ framework.

The CIs for the entropy measures of the EGP distribution can be constructed using the delta method. For any entropy function H(f), the approximate variance is given by

$$\tilde{V}\left(\tilde{H}(f)\right)={\left[V^{T}I^{-1}\left(\Theta \right)V\right]|}_{\Theta =\Theta_{es}},$$

where$\;V^{T}=\left(\frac{\partial H(f)}{\partial \alpha },\;\frac{\partial H(f)}{\partial \beta },\;\frac{\partial H(f)}{\partial \theta },\;\frac{\partial H(f)}{\partial \lambda }\right)$ and $I^{-1}\left(\Theta \right)\;$is the inverse Fisher information matrix. Here, H(f) may correspond to the Shannon, Rényi, or Tsallis entropy of the EGP distribution. Accordingly, the standardized statistic $\frac{\tilde{H}\left(f\right)-H\left(f\right)}{\sqrt{\tilde{V}\left(\tilde{H}(f)\right)}}$ is asymptotically distributed as N (0, 1). Thus, the approximate 100(1−ω)% delta CIs for H(f) can be expressed as

$$\tilde{H}\left(f\right)\pm Z_{\frac{\omega }{2}}\;\sqrt{\tilde{V}\left(\tilde{H}(f)\right)},$$

where $Z_{\frac{\omega }{2}}$ is the upper $\frac{\omega }{2}\;$percentile of the standard normal distribution.

For further details on the delta method and its applications in entropy estimation, one may refer to [9].

It is noteworthy that the ML estimators of the entropy measures for the EGP distribution under the special cases OS and ROS, as well as the UR and LR values, can be readily obtained by substituting the corresponding parameter estimators from each sampling scheme into the respective entropy formulations. Since these estimators are derived directly from the general GOS/DGOS framework, this provides a unified procedure for parameter and entropy estimation across all ordered sampling schemes.

**Several notable points can be highlighted:**

- For complete samples including OS, ROS, or unordered data, the ML estimators of the parameters coincide, as their likelihoods differ only by constants, leaving the Shannon, Rényi, and Tsallis entropy estimators unaffected by data ordering.
- In contrast, incomplete or selectively observed data, such as censored or record-based schemes, produce distinct likelihoods and parameter estimators, as omitted observations alter the weighting of survival and cumulative functions, introducing variability in the estimators, particularly for small or skewed samples.
- Special cases, such as LR and UR values, rely solely on extreme observations, which reduces the efficiency of entropy estimation.

## 4. A Numerical Study

### 4.1. Simulation Study

This section presents a comprehensive Monte Carlo simulation study to assess the finite-sample performance of the proposed estimation procedures for the EGP distribution under different ordered-data structures. The primary objective is to evaluate the accuracy, stability, and robustness of parameter and entropy estimation when inference is conducted using GOS and their dual forms, DGOS. Random samples were generated from the EGP distribution for various combinations of sample sizes and true parameter values to reflect different distributional shapes and tail behaviors. For each configuration, a number, N=1000, of Monte Carlo replications were performed to ensure a reliable empirical assessment. Four representative parameter settings of the EGP distribution were considered, namely, (α=0.6,β=1.3,θ=0.7,λ=0.8), (α=0.7,β=1.8,θ=1.1,λ=0.5), (α=0.3,β=1.6,θ=0.9,λ=1.3), and (α=3.3, β=1.8,θ=0.4,λ=1.9), as specified in Table 3, Table 4, Table 5, Table 6 and Table 7. These settings were selected to represent different parameter magnitudes and distributional behaviors of the EGP model, thereby providing representative scenarios for evaluating the finite-sample performance of the proposed estimation procedures.

To reflect the proposed inferential framework, the simulation study was conducted under representative special cases of the GOS and DGOS frameworks. Specifically, three sampling structures were considered:

- Complete sample: All generated observations are fully observed and utilized in the estimation process.
- UR values: Observations exceeding all previous values are sequentially retained.
- LR values: Observations smaller than all previous values are retained.

This design enables a direct comparison of estimation performance under different ordered-data structures within a unified inferential setting. Each structure was treated as an independent inference framework, with estimation performed using only the information retained under the respective models. For each simulated dataset and ordered-data scheme, parameters were estimated via the ML method. Based on these estimates, the entropy measures were computed for selected values of the associated tuning parameters. Standard errors were derived from the observed Fisher information matrix, and asymptotic CIs were constructed for both parameters and entropy measures. For entropy measures, CIs were derived using the delta method. The entire procedure was repeated across all Monte Carlo replications. Estimator performance was evaluated using the *mean squared error* (MSE), *relative error* (RE), and *coverage probability* (CP).

The simulation results presented in Table 3, Table 4, Table 5, Table 6 and Table 7 indicate that:

- For the complete-sample case, the MSE, RE, and CI lengths generally decrease as the sample size increases, indicating improved estimation accuracy and supporting the asymptotic consistency of the estimates. In addition, the CP remains reasonably close to the nominal confidence level.
- For the LR and UR data structures, the estimates display comparatively higher variability due to the reduced amount of information retained. Nevertheless, the estimation accuracy improves consistently as the number of record observations increases. The results further show that entropy measures are generally more sensitive to the underlying ordered data structure than the model parameters, particularly for LR samples.

Overall, the simulation findings demonstrate that GOS and their dual forms, through the special cases of LR and UR values, substantially affect the estimation behavior of entropy measures for the EGP distribution. While complete samples provide the most stable estimation results, the relative performance of the LR and UR frameworks varies across different parameter settings and entropy measures, reflecting the sensitivity of entropy-based inference to the underlying record structure.

**Key Findings from the Simulation Study**

- Effect of sample size:

For all sampling structures, the MSE and RE decrease as the sample size increases, indicating improved estimation accuracy and stability. For the complete-sample case, the confidence interval lengths also decrease with increasing sample size, reflecting the asymptotic consistency and efficiency of the ML estimators.

- Coverage probability:

For the complete-sample scheme, the coverage probabilities remain close to the nominal confidence level across all simulation settings, demonstrating the satisfactory finite-sample performance of the proposed asymptotic CIs.

- Effect of the sampling structure:

The complete sample scheme consistently provides the most accurate estimates because it utilizes all available observations. In contrast, the UR and LR schemes retain only record observations, leading to a reduction in the effective sample size and consequently larger estimation errors and greater estimation variability.

- Comparison between UR and LR schemes:

Although both record-based sampling schemes exhibit lower estimation efficiency than the complete-sample scheme, their performance improves steadily as the number of retained record observations increases. In most simulation settings, the LR scheme shows slightly greater variability in entropy estimation than the UR scheme, indicating that entropy estimation is more sensitive to lower-record information.

- Overall performance:

The simulation results demonstrate that the proposed estimation procedure performs satisfactorily under all three sampling structures. The estimation accuracy improves consistently as the amount of available information increases, confirming the robustness and effectiveness of the proposed methodology.

**Remark:**

Under the UR and LR sampling schemes, inference is based solely on record observations, resulting in a substantially smaller effective sample size. Consequently, the asymptotic normal approximation underlying the Wald CIs may be less reliable than in the complete sample case. Therefore, the corresponding CIs should be interpreted with caution, while the performance of the estimators is primarily assessed using the point-estimation measures, namely, the MSE and RE.

### 4.2. Real Data Analysis

In this section, two real datasets arising from lifetime and economic applications are analyzed to evaluate the adequacy, flexibility, and empirical performance of the proposed EGP distribution. The primary objective is to examine the flexibility of the proposed model and investigate the impact of GOS and DGOS on parameter estimation and entropy measures across different ordered data schemes.

The analysis is conducted under four data structuring mechanisms: OS, ROS, UR, and LR. Specifically, OS and UR represent special cases of the GOS framework, whereas ROS and LR represent special cases of the DGOS framework. Accordingly, inference on the EGP distribution parameters, together with their *standard errors* (SEs) and CIs, can be carried out within a unified inferential framework. This setting also enables a coherent comparison of entropy estimates derived from different ordered-data representations. In practice, the choice of a particular GOS or DGOS structure should be guided by the mechanism through which the data are collected rather than by the fitted distribution itself. Ordered complete samples (OS or ROS) are appropriate when all observations are available, whereas record-based structures such as UR and LR are suitable when only successive upper or lower record observations are naturally retained during the data collection process. Consequently, the adopted ordered-data structure should reflect the information available in the application under consideration.

#### 4.2.1. Dataset I: Lifetime Data

The first dataset consists of thirty observed failure times corresponding to the lifetimes of a certain device, as reported by [48]. These data originate from controlled reliability experiments and have been employed in the literature to evaluate the adequacy of lifetime distribution models. For this dataset, inference is carried out with OS and UR schemes, both of which arise naturally as special cases of the GOS framework. The observed lifetimes are given by

0.0094, 0.05, 0.4064, 4.6307, 5.1741, 5.8808, 6.3348, 7.1645, 7.2316, 8.2604, 9.2662, 9.3812, 9.5223, 9.8783, 9.9346, 10.019, 10.408, 10.479, 11.076, 11.325, 11.528, 11.923, 12.029, 12.074, 12.184, 12.355, 12.538, 12.805, 13.462, and 13.853.

#### 4.2.2. Dataset II: Economic Data

The second dataset represents economic inequality data, namely, the poverty gap at USD 3.00 per day (2021 PPP), expressed as a percentage of the population. The data was obtained from the World Bank Open Data repository. This dataset is analyzed under ROS and LR schemes, which are special cases of the DGOS framework. Such data structures are particularly relevant in economic studies where interest often focuses on declining trends or lower-tail behavior.

The observed values are given by

43.1, 41.1, 38.6, 34.8, 32.6, 31.2, 29.3, 26.3, 31, 30, 29.3, 28.1, 27, 23.9, 21.6, 19.6, 19.6, 20.4, 18.7, 17.6, 16.4, 14.4, 12.4, 10.7, 8.8, 8.5, 7.5, 7, 6.2, 5.3, 4.2, 3.5, 1.9, 1.6, 1.2, 1, 0.8, 0.7, 0.6, 0.6, 0.6, 0.5, 0.5, and 0.4 [49].

**Figure 3:**
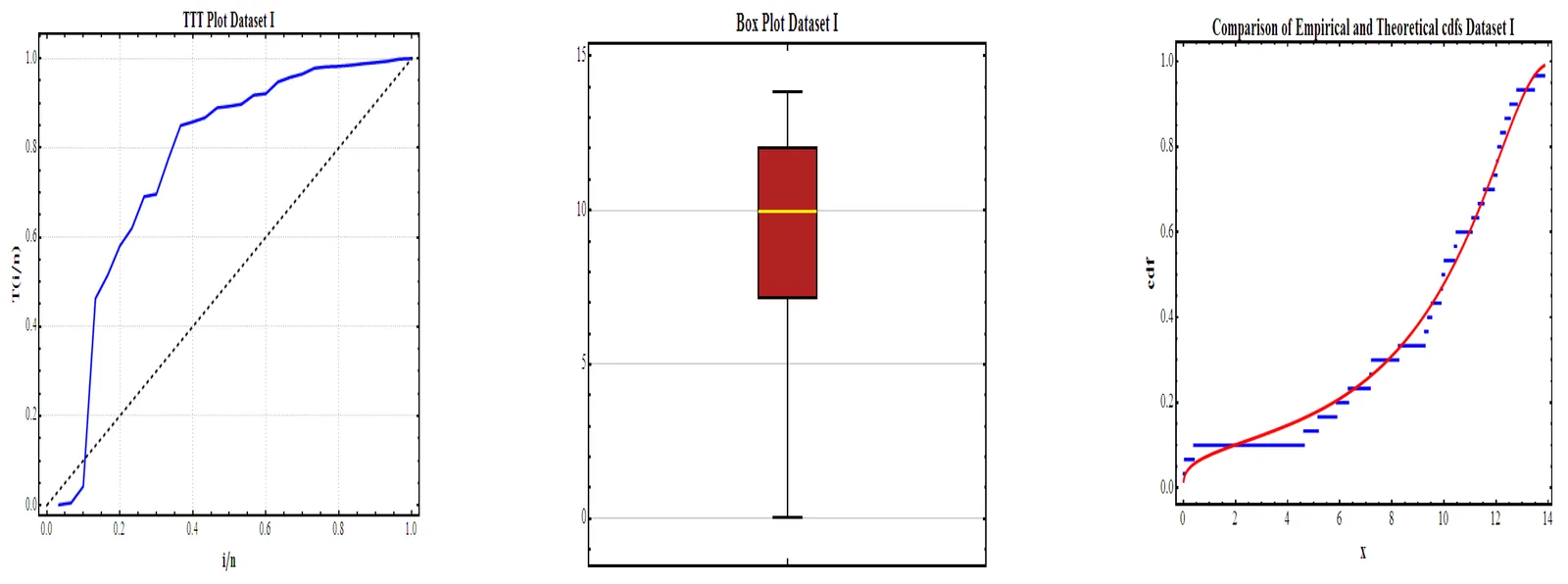
TTT plot, boxplot, and comparison of the empirical and fitted cdfs for Dataset I.

**Figure 4:**
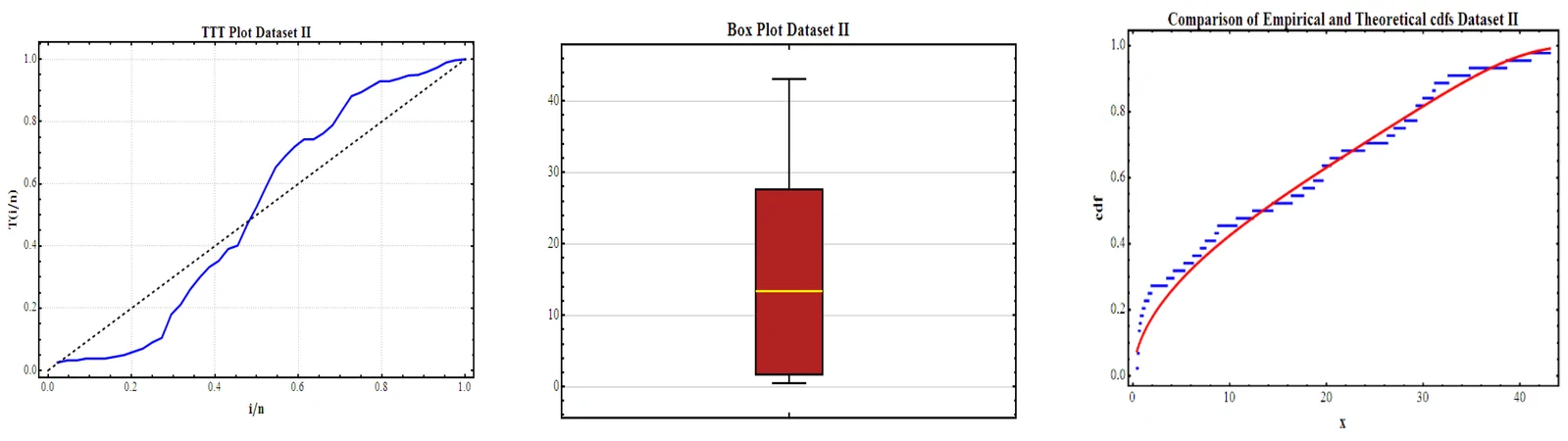
TTT plot, boxplot, and comparison of the empirical and fitted cdfs for Dataset II.

To further explore the underlying failure behavior of the data, the *Total Time on Test* (TTT) transform proposed by [50] is employed. The corresponding TTT plots, along with the associated boxplots for Datasets I and II, are presented in Figure 3 and Figure 4, respectively. According to [50], the concave TTT curve lying above the diagonal for Dataset I indicates a decreasing hrf. In contrast, the TTT curve for Dataset II crosses the diagonal, suggesting a bathtub hrf. These heterogeneous failure patterns emphasize the flexibility of the EGP distribution in accommodating complex hazard rate behaviors.

To assess the goodness of fit of the proposed model, the Kolmogorov–Smirnov (KS) test is applied to both datasets, and the resulting KS statistics and corresponding *p*-values are reported in Table 8. In addition, several information-criterion measures are considered, including the Akaike information criterion (AIC), the consistent Akaike information criterion (CAIC), the Bayesian information criterion (BIC), and the Hannan–Quinn information criterion (HQIC). For all these criteria, smaller values indicate a better model fit. For Dataset I, the proposed EGP distribution is compared with several well-known competing models, namely, the exponentiated generalized exponential–geometric (EGEG), exponentiated generalized exponential-exponential (EG-EE), the new extended Chen (NEC) distribution proposed by [51], and the Maxwell distribution. For Dataset II, the competing models include the exponentiated generalized Weibull–exponential (EGWE), P, EGEG, NEC, Lomax, Fréchet, and Maxwell distributions.

The results summarized in Table 8 show that the EGP distribution attains the smallest values of the considered criteria for both datasets, thereby confirming its empirical adequacy and competitive performance. This conclusion is further supported by the close agreement between the empirical and fitted cumulative distribution functions displayed in Figure 3 and Figure 4. Figure 3 and Figure 4 present the TTT plots, boxplots, and comparisons between the empirical and fitted cumulative distribution functions for Datasets I and II, respectively. In both datasets, the TTT plots indicate non-constant hazard rate behavior, supporting the use of a flexible lifetime model such as the EGP distribution. The boxplots provide concise summaries of the distributions, illustrating the spread and central tendency of the observations without revealing extreme outliers. This conclusion is further reinforced by the close agreement between the empirical and fitted cumulative distribution functions, which demonstrates that the EGP distribution provides an adequate fit across the entire range of observations for both datasets and accurately captures their underlying distributional characteristics.

**Figure 5:**
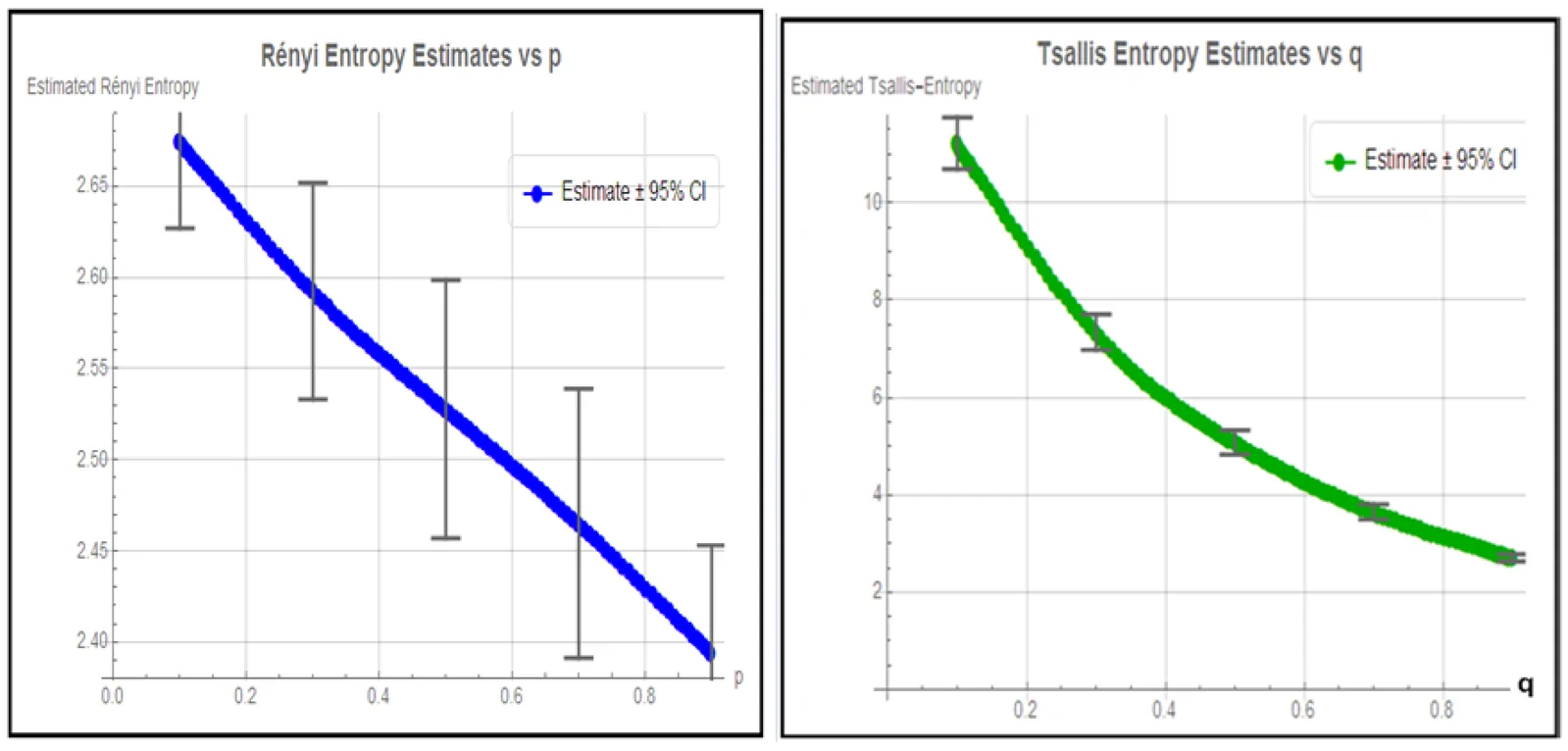
Rényi and Tsallis entropy estimates with CIs for selected (p, q) values based on OS for Dataset I.

**Figure 6:**
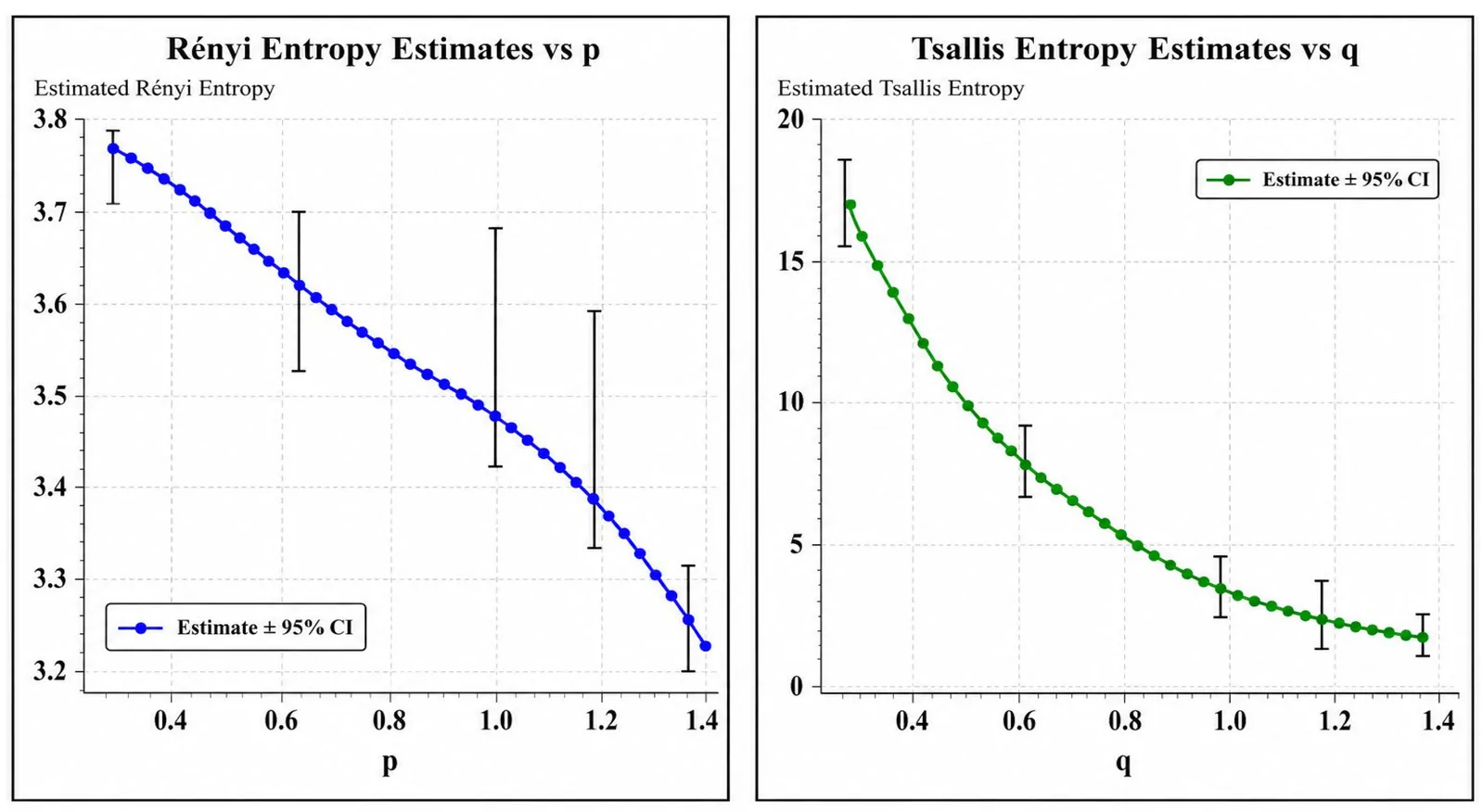
Rényi and Tsallis entropy estimates with CIS for selected (p, q) values based on ROS for Dataset II.

With Dataset I, estimates of parameters and entropies were performed using OS and UR. ML estimates of both the model parameters and entropy measures, along with SE and CIS, are reported in Table 9. Entropy estimates together with their corresponding 95% confidence intervals are also illustrated in Figure 5 for Dataset I. Figure 5 depicts the estimated Rényi and Tsallis entropies for selected values of the entropy orders based on the original sample. The estimates vary smoothly with the entropy order, reflecting the expected dependence of these measures on their tuning parameters. The associated confidence intervals quantify the estimation uncertainty and indicate reasonably stable estimation across the selected values, supporting the practical applicability of the proposed estimation procedure. For Dataset II, a parallel analysis was conducted using the ROS and LR sampling schemes, with the corresponding results reported in Table 10 and illustrated in Figure 6. Figure 6 presents the estimated Rényi and Tsallis entropies together with their 95% confidence intervals for selected entropy orders. Similar to Dataset I, the entropy estimates exhibit systematic variation with the entropy order while maintaining consistent trends. The confidence intervals provide an assessment of the estimation precision and demonstrate that the proposed inferential procedure yields stable entropy estimates under the record sampling schemes. Since the model parameters are strictly positive, any negative lower confidence limit obtained from the Wald approximation was truncated to zero when reporting the CIs in Table 9 and Table 10.

The analysis demonstrates that the EGP distribution provides an excellent fit to both datasets, as confirmed by the ML estimates and associated goodness-of-fit measures. Notably, the selection of an ordered data structure (OS, ROS, UR, or LR) substantially influences parameter and entropy estimates. These results highlight the pivotal role of GOS and their duals in entropy-based inference, particularly for records or censored samples, where partial observations can markedly impact the estimation of both the model parameters and the entropy measures.

In the TTT plot, the solid blue line represents the empirical values, whereas the dashed black line represents the theoretical reference line. In the cdf plot, the blue step line represents the empirical cdf, while the red solid line represents the fitted theoretical cdf.

**Results and Practical Interpretation**

The results presented in Table 8, Table 9 and Table 10 demonstrate that the proposed EGP distribution provides the best overall fit for both datasets, as evidenced by the smallest values of the adopted goodness-of-fit criteria. This finding is further supported by the close agreement between the empirical and fitted cumulative distribution functions shown in Figure 4 and Figure 5, confirming the flexibility of the proposed model in describing datasets from different application domains.

For Dataset I, which consists of device failure times, the estimated entropy provides a meaningful measure of the uncertainty associated with the lifetime distribution. From an information-theoretic perspective, larger entropy values indicate greater uncertainty in the occurrence of failures, whereas smaller values correspond to a more concentrated lifetime distribution. Since the EGP distribution achieves the best fit, the corresponding entropy estimates are expected to provide a reliable characterization of the uncertainty underlying the failure mechanism, making the proposed model useful for reliability assessment and maintenance planning.

For Dataset II, representing poverty-gap data, the estimated entropy reflects the uncertainty and heterogeneity in the distribution of poverty intensity. The superior performance of the proposed EGP distribution suggests that it captures the underlying distribution more accurately than the competing models. Consequently, the resulting entropy estimates provide a meaningful summary of the uncertainty inherent in the economic data, which may support socioeconomic analysis and policy evaluation.

**Summary of the Real Data Results**

1. Model adequacy: The proposed EGP distribution provides the best fit for both datasets, as evidenced by the smallest goodness-of-fit statistics and the close agreement between the empirical and fitted cumulative distribution functions.
2. Practical significance of entropy: The estimated entropy values quantify the uncertainty associated with the underlying probability distribution. In Dataset I, entropy characterizes the uncertainty in device lifetimes, whereas in Dataset II, it reflects the uncertainty and heterogeneity in poverty-gap values.
3. Effect of the sampling structure: The adopted sampling scheme (OS, UR, ROS, or LR) noticeably affects both parameter and entropy estimation, highlighting the importance of the sampling structure in entropy-based inference.
4. Effect of the entropy parameters: The estimated Rényi and Tsallis entropies decrease as the entropy parameters p and q increase, indicating the influence of these parameters on the entropy measure.
5. Negative Shannon entropy estimates: Some estimated Shannon entropy values are negative. This is mathematically valid for continuous distributions and reflects relatively more concentrated probability distributions rather than negative uncertainty.
6. Practical Implications: The real data analyses illustrate that the proposed EGP distribution can successfully model datasets from both reliability and socioeconomic applications while providing entropy measures that offer meaningful insight into the uncertainty associated with the observed data.

## 5. Conclusions

In this paper, we proposed a new flexible lifetime model, namely, the EGP distribution, by combining the Pham distribution with the exponentiated generalized family. The proposed model exhibits considerable flexibility in modeling a wide variety of data behaviors, as evidenced by its ability to generate different shapes of the pdf and hrf, including non-standard patterns such as non-monotonic decreasing and inverted Vtub shapes. A unified inferential framework was established by employing GOS and their dual (DGOS), allowing for a comprehensive analysis under multiple sampling schemes, including OS, ROS, and UR and LR values. Within this framework, ML estimation was utilized for parameter estimation, and entropy measures, namely, Shannon, Rényi, and Tsallis, were obtained via the invariance property. The delta method was applied to construct approximate CIs for the entropy measures.

The results revealed that the choice of sampling scheme has a substantial impact on the estimation accuracy. Full-sample approaches (OS and ROS) yield more reliable and efficient estimates, whereas record-based schemes (UR and LR) lead to increased variability due to reduced information content. The superiority of the proposed model was further confirmed through real data applications, where it outperformed several competing models according to goodness-of-fit criteria. In conclusion, the EGP distribution, combined with the GOS or DGOS framework, provides a powerful and unified approach for modeling and inference in reliability and lifetime data analyses, particularly when dealing with ordered or partially observed data.

The findings of this study provide a foundation for several directions of future research. Although the present study is confined to inference within the GOS and DGOS frameworks and is illustrated through complete and record-based sampling schemes, the proposed methodology may be extended to more general sampling settings. In particular, future work may consider entropy estimation under progressive, Type-II, and hybrid censoring schemes, as well as the development of Bayesian and robust inferential procedures for the EGP distribution and its associated entropy measures. Furthermore, extending the proposed framework to other generalized lifetime distributions and more complex reliability models represent a promising direction for future investigation.

## Figures and Tables

**Table 1:** Numerical values of E(X), Var(X), Sk(X), Ku(X), and γ of the EGP distribution for selected values of the parameters.

			θ = 0.4					θ = 2.4					θ = 6.5				
α	β	λ	E(X)	Var(X)	Sk(X)	Ku(X)	γ	E(X)	Var(X)	Sk(X)	Ku(X)	γ	E(X)	Var(X)	Sk(X)	Ku(X)	γ
**0.6**	**1.5**	**0.8**	5.037	24.93	1.255	4.315	0.991	0.740	1.077	2.483	11.21	1.402	0.191	0.096	3.325	19.35	1.624
		**2**	8.625	27.55	0.681	3.176	0.609	1.403	1.643	1.657	6.670	0.913	0.374	0.163	2.314	11.09	1.080
		**4**	11.56	24.10	0.481	3.083	0.429	2.064	1.942	1.290	5.284	0.675	0.569	0.215	1.852	8.319	0.814
	**3.5**	**0.8**	0.769	0.580	1.255	4.315	0.991	0.113	2.483	2.483	11.21	1.402	0.029	0.002	3.325	19.35	1.624
		**2**	1.316	0.641	0.681	3.176	0.609	0.214	0.038	1.657	6.670	0.913	0.057	0.004	2.314	11.09	1.080
		**4**	1.764	0.573	0.481	3.083	0.429	0.315	0.045	1.290	5.284	0.675	0.087	1.852	1.852	8.319	0.814
	**5.5**	**0.8**	0.460	0.208	1.255	4.315	0.991	0.068	0.009	2.483	11.21	1.402	0.017	0.001	3.325	19.35	1.624
		**2**	0.787	0.230	0.681	3.176	0.609	0.128	0.014	1.657	6.670	0.913	0.034	0.001	2.314	11.09	1.080
		**4**	1.056	0.205	0.481	3.083	0.429	0.188	0.016	1.290	5.284	0.675	0.052	0.002	1.852	8.319	0.814
**2.8**	**1.5**	**0.8**	1.247	0.155	−0.573	2.727	0.316	0.775	0.091	−0.072	2.383	0.389	0.565	0.054	0.105	2.477	0.413
		**2**	1.525	0.060	−0.753	3.693	0.160	0.995	0.050	−0.245	2.808	0.225	0.736	0.034	−0.034	2.760	0.250
		**4**	1.660	0.028	−0.572	3.560	0.102	1.122	0.030	−0.200	2.932	0.155	0.840	0.023	0.002	2.878	0.179
	**3.5**	**0.8**	0.834	0.069	−0.573	2.727	0.316	0.518	0.041	−0.072	2.383	0.389	0.377	0.024	0.105	2.477	0.413
		**2**	1.019	0.027	−0.753	3.693	0.160	0.665	0.022	−0.245	2.808	0.225	0.492	0.015	−0.034	2.760	0.250
		**4**	1.101	0.013	−0.572	3.560	0.102	0.750	0.014	−0.200	2.932	0.155	0.562	0.010	0.002	2.878	0.179
	**5.5**	**0.8**	0.747	0.056	−0.573	2.727	0.316	0.464	0.033	−0.072	2.383	0.389	0.338	0.020	0.105	2.477	0.413
		**2**	0.913	0.021	−0.753	3.693	0.160	0.596	0.018	−0.245	2.808	0.225	0.441	0.012	−0.034	2.760	0.250
		**4**	0.994	0.010	−0.572	3.560	0.102	0.672	0.011	−0.200	2.932	0.155	0.503	0.008	0.002	2.878	0.179
**4.7**	**1.5**	**0.8**	1.124	0.054	−0.975	3.741	0.207	0.841	0.045	−0.497	2.854	0.251	0.695	0.034	−0.344	2.748	0.264
		**2**	1.282	0.016	−1.017	4.596	0.099	0.991	0.019	−0.528	3.257	0.139	0.827	0.016	−0.336	3.031	0.153
		**4**	1.351	0.007	−0.727	3.990	0.062	1.068	0.010	−0.395	3.186	0.094	0.898	0.009	−0.214	3.008	0.108
	**3.5**	**0.8**	0.884	0.034	−0.975	3.741	0.207	0.661	0.028	−0.497	2.854	0.251	0.547	0.021	−0.344	2.748	0.264
		**2**	1.008	0.010	−1.017	4.596	0.099	0.779	0.012	−0.528	3.257	0.139	0.650	0.010	−0.336	3.031	0.153
		**4**	1.063	0.004	−0.727	3.990	0.062	0.840	0.006	−0.395	3.186	0.094	0.706	0.006	−0.214	3.008	0.108
	**5.5**	**0.8**	0.828	0.029	−0.975	3.741	0.207	0.620	0.024	−0.497	2.854	0.251	0.512	0.018	−0.344	2.748	0.264
		**2**	0.944	0.009	−1.017	4.596	0.099	0.730	0.010	−0.528	3.257	0.139	0.609	0.009	−0.336	3.031	0.153
		**4**	0.995	0.004	−0.727	3.990	0.062	0.787	0.005	−0.395	3.186	0.094	0.662	0.005	−0.214	3.008	0.108

**Table 2:** Numerical values of entropies of the EGP distribution for selected combinations of the parameters.

			θ = 0.4					θ = 2.4					θ = 6.5				
α	β	λ	$HR_{0.2}$	$HR_{1.2}$	$HT_{0.4}$	$HT_{1.4}$	HS	$HR_{0.2}$	$HR_{1.2}$	$HT_{0.4}$	$HT_{1.4}$	HS	$HR_{0.2}$	$HR_{1.2}$	$HT_{0.4}$	$HT_{1.4}$	HS
**0.6**	**1.5**	**0.8**	3.257	2.356	8.327	1.447	2.512	1.954	0.122	2.248	−0.250	0.435	0.940	−1.384	0.251	−2.622	−1.015
		**2**	3.371	2.960	9.628	1.727	2.992	2.123	1.259	3.124	0.953	1.332	1.127	−0.089	0.743	−0.177	0.013
		**4**	3.395	2.955	9.771	1.724	2.993	2.216	1.529	3.582	1.121	1.583	1.242	0.293	1.051	0.226	0.364
	**3.5**	**0.8**	1.377	0.4763	1.568	0.265	0.632	0.074	−1.758	−0.400	−3.335	−1.445	−0.940	−3.264	−1.046	−8.366	−2.895
		**2**	1.490	1.079	1.989	0.860	1.112	0.243	−0.621	−0.116	−0.782	−0.548	−0.753	−1.969	−0.887	−3.178	−1.867
		**4**	1.515	1.075	2.035	0.853	1.113	0.336	−0.351	0.032	−0.426	−0.297	−0.638	−1.588	−0.787	−2.323	−1.516
	**5.5**	**0.8**	0.863	−0.038	0.710	−0.244	0.119	−0.440	−2.272	−0.736	−4.665	−1.959	−1.453	−3.777	−1.210	−10.843	−3.408
		**2**	0.977	0.566	1.020	0.486	0.599	−0.270	−1.135	−0.527	−1.530	−1.061	−1.267	−2.483	−1.093	−4.473	−2.380
		**4**	1.001	0.561	1.054	0.478	0.600	−0.177	−0.864	−0.418	−1.093	−0.811	−1.151	−2.101	−1.020	−3.423	−2.030
**2.8**	**1.5**	**0.8**	0.661	0.391	0.683	0.338	0.424	0.421	0.175	0.365	0.149	0.201	0.204	−0.080	0.097	−0.105	−0.052
		**2**	0.422	−0.089	0.240	−0.130	−0.041	0.284	−0.124	0.129	−0.161	−0.086	0.102	−0.315	−0.063	−0.372	−0.277
		**4**	0.142	−0.434	−0.101	−0.519	−0.387	0.105	−0.375	−0.092	−0.444	−0.333	−0.041	−0.519	−0.223	−0.618	−0.478
	**3.5**	**0.8**	0.258	−0.012	0.179	−0.040	0.021	0.018	−0.228	−0.071	−0.262	−0.202	−0.198	−0.483	−0.282	−0.561	−0.455
		**2**	0.019	−0.492	−0.170	−0.590	−0.444	−0.119	−0.527	−0.257	−0.626	−0.489	−0.301	−0.718	−0.407	−0.874	−0.680
		**4**	−0.261	−0.837	−0.437	−1.047	−0.789	−0.298	−0.778	−0.430	−0.958	−0.736	−0.444	−0.922	−0.533	−1.163	−0.881
	**5.5**	**0.8**	0.148	−0.122	0.061	−0.154	−0.089	−0.092	−0.338	−0.173	−0.386	−0.312	−0.308	−0.593	−0.370	−0.699	−0.565
		**2**	−0.091	−0.602	−0.265	−0.729	−0.554	−0.229	−0.637	−0.347	−0.766	−0.599	−0.411	−0.828	−0.488	−1.026	−0.790
		**4**	−0.371	−0.9470	−0.516	−1.206	−0.899	−0.408	−0.888	−0.509	−1.114	†DC	−0.554	−1.032	−0.606	−1.328	−0.991
**3.**	**1.5**	**0.8**	0.402	0.037	0.306	0.001	0.079	0.254	−0.042	0.155	−0.069	−0.011	0.103	−0.210	−0.013	−0.248	−0.177
		**2**	0.078	−0.505	−0.150	−0.610	−0.454	0.035	−0.424	−0.143	−0.502	−0.383	−0.081	−0.533	−0.244	−0.635	−0.492
		**4**	−0.256	−0.873	−0.451	−1.100	−0.824	−0.198	−0.709	−0.367	−0.866	†DC	−0.276	−0.775	−0.422	−0.956	−0.733
	**3.5**	**0.8**	0.106	−0.260	−0.016	−0.314	−0.218	−0.043	−0.339	−0.142	−0.393	−0.307	−0.194	−0.506	−0.283	−0.594	−0.474
		**2**	−0.219	−0.802	−0.397	−1.002	−0.751	−0.262	−0.721	−0.392	−0.880	−0.680	−0.378	−0.830	−0.476	−1.030	−0.789
		**4**	−0.553	−1.170	−0.650	−1.554	−1.121	−0.495	−1.006	−0.579	−1.290	−0.963	−0.573	−1.072	−0.625	−1.391	†DC
	**5.5**	**0.8**	0.025	−0.341	−0.094	−0.406	−0.299	−0.124	−0.420	−0.215	−0.489	−0.388	−0.275	−0.588	−0.348	−0.696	−0.555
		**2**	−0.300	−0.883	−0.458	−1.118	−0.832	−0.343	−0.802	−0.452	−0.992	−0.761	−0.459	−0.911	−0.533	−1.147	−0.870
		**4**	−0.634	−1.251	−0.698	−1.687	†DC	−0.576	−1.087	−0.631	−1.415	−1.044	−0.654	−1.153	−0.675	−1.519	−1.111

†DC: The numerical integration does not converge.

**Table 3:** Average values, MSEs, REs, 95% CIs, and CPs of the ML estimates for the parameters of EGP distribution under the complete sample framework (α=0.6,β=1.3,θ=0.7, and λ=0.8).

n	Θ	Average	MSE	RE	CI			CP
					L	U	Length	
**30**	α	0.6305	0.0047	0.1140	0.5099	0.7511	0.2412	0.9000
	β	1.2946	0.0024	0.0374	1.1994	1.3898	0.1904	0.9150
	θ	0.6663	0.0169	0.1858	0.4187	0.9139	0.4952	0.9110
	λ	0.7988	0.0163	0.1594	0.5474	1.0502	0.5028	0.9330
**80**	α	0.6091	0.0033	0.0956	0.4975	0.7207	0.2232	0.9440
	β	1.2998	0.0023	0.0370	1.2050	1.3945	0.1895	0.9300
	θ	0.6954	0.0148	0.1740	0.4556	0.9353	0.4797	0.9120
	λ	0.8023	0.0134	0.1450	0.5738	1.0308	0.4570	0.9380
**120**	α	0.6142	0.0026	0.0857	0.5168	0.7116	0.1948	0.9480
	β	1.2969	0.0016	0.0305	1.2190	1.3748	0.1558	0.9490
	θ	0.6788	0.0118	0.1552	0.4688	0.8888	0.4200	0.9250
	λ	0.7907	0.0112	0.1321	0.5833	0.9981	0.4148	0.9460

**Table 4:** Average values, MSEs, REs, 95% CIs, and CPs of the ML estimates for the entropies of the EGP distribution under the complete-sample framework (α=0.6, β=1.3,θ=0.7, and λ=0.8).

n	Entropy	Average	MSE	RE	CI			CP
					L	U	Length	
**30**	${HR}_{0.5}$	2.5219	0.0377	0.0743	2.1763	2.8675	0.6912	0.8750
	${HR}_{0.9}$	2.3579	0.0232	0.0620	2.1181	2.5977	0.4796	0.9160
	${HT}_{0.5}$	5.0820	0.4138	0.1193	3.9400	6.2239	2.2839	0.8890
	${HT}_{0.9}$	2.6600	0.0368	0.0690	2.3583	2.9617	0.6034	0.9180
**80**	${HR}_{0.5}$	2.5902	0.0098	0.0379	2.3967	2.7837	0.3870	0.8840
	${HR}_{0.9}$	2.4610	0.0051	0.0290	2.3175	2.6044	0.2869	0.9370
	${HT}_{0.5}$	5.3111	0.1341	0.0679	4.5914	6.0308	1.4394	0.8960
	${HT}_{0.9}$	2.7906	0.0083	0.0328	2.6065	2.9746	0.3681	0.9370
**120**	${HR}_{0.5}$	2.5771	0.0054	0.0280	2.4474	2.7069	0.2595	0.9090
	${HR}_{0.9}$	2.4468	0.0038	0.0251	2.3237	2.5699	0.2462	0.9450
	${HT}_{0.5}$	5.2588	0.0700	0.0489	4.7895	5.7282	0.9387	0.9170
	${HT}_{0.9}$	2.7723	0.0061	0.0282	2.6154	2.9292	0.3138	0.9410

**Table 5:** Average values, MSEs, and REs of the ML estimates for the parameters of EGP distribution under UR and LR values (α=0.7,β=1.8,θ=1.1, and λ=0.5).

r	Θ	LR			UR		
		Average	MSE	RE	Average	MSE	RE
**5**	α	0.8420	0.0359	0.2707	0.8660	0.0629	0.3584
	β	1.9953	0.0657	0.1425	1.9026	0.0537	0.1288
	θ	1.2631	0.1089	0.3000	1.1491	0.0702	0.2409
	λ	0.5701	0.0200	0.2830	0.6213	0.0570	0.4776
**7**	α	0.8623	0.0345	0.2655	0.7981	0.0300	0.2476
	β	1.9891	0.0651	0.1418	1.9059	0.0426	0.1146
	θ	1.2103	0.0928	0.2769	1.1836	0.0686	0.2381
	λ	0.5907	0.0164	0.2567	0.5636	0.05686	0.4769
**9**	α	0.8388	0.0237	0.2199	0.7753	0.0187	0.1954
	β	1.9231	0.0665	0.1433	1.9116	0.0229	0.0840
	θ	1.1819	0.0879	0.2696	1.2214	0.0431	0.1888
	λ	0.5913	0.0107	0.2069	0.5162	0.0385	0.3925

**Table 6:** Average values, MSEs, and REs of the ML estimates for the parameters of EGP distribution under UR and LR values (α=0.3,β=1.6,θ=0.9, and λ=1.3).

r	Θ	LR			UR		
		Average	MSE	RE	Average	MSE	RE
**5**	α	0.3624	0.0072	0.2835	0.3376	0.0051	0.2395
	β	1.7487	0.0501	0.1399	1.6393	0.0113	0.0666
	θ	1.0014	0.0714	0.2970	0.8441	0.0449	0.2355
	λ	1.4001	0.0821	0.2204	1.7465	0.2032	0.3005
**7**	α	0.3642	0.0065	0.2706	0.3417	0.0040	0.2122
	β	1.7159	0.0438	0.1308	1.6407	0.0059	0.0483
	θ	0.9038	0.0621	0.2770	0.8615	0.0314	0.1969
	λ	1.4737	0.0729	0.2078	1.4405	0.1778	0.3243
9	α	0.3496	0.0056	0.2510	0.3295	0.0030	0.1848
	β	1.6220	0.0251	0.0991	1.6669	0.0129	0.0712
	θ	0.8641	0.0370	0.2139	0.9024	0.0156	0.1390
	λ	1.4448	0.0502	0.1724	1.3499	0.1555	0.3033

**Table 7:** Average values and MSEs of the ML estimates for the entropies of EGP distribution under UR and LR values (α=3.3, β=1.8,θ=0.4, and λ=1.9).

r	Entropy	LR		UR	
		Average	MSE	Average	MSE
**5**	$HR_{0.3}$	0.0177	0.0462	0.0044	0.0460
	$HR_{0.6}$	0.2528	0.2481	0.2171	0.2499
	${HT}_{0.3}$	0.0330	0.0388	0.0198	0.0412
	${HT}_{0.6}$	0.2754	0.2685	0.2421	0.2635
**7**	$HR_{0.3}$	0.0082	0.0356	0.0061	0.0361
	$HR_{0.6}$	0.0355	0.1633	0.1232	0.1716
	${HT}_{0.3}$	0.0203	0.0324	0.0184	0.0339
	${HT}_{0.6}$	0.0566	0.1581	0.1402	0.1817
9	$HR_{0.3}$	0.0668	0.0266	0.0581	0.0192
	$HR_{0.6}$	0.0150	0.1529	0.0912	0.1508
	${HT}_{0.3}$	0.0769	0.0264	0.0655	0.0198
	${HT}_{0.6}$	0.0359	0.1506	0.1065	0.1583

**Table 8:** Goodness-of-fit statistics (KS, *p*-value, AIC, BIC, CAIC, HQIC) for competing models to both datasets.

Datasets	Model	KS	*p*-Value	AIC	BIC	CAIC	HQIC
**Dataset I**	**EGP**	0.0725	0.9974	147.1334	152.7381	148.7333	148.9264
	EGEG	0.0924	0.9597	148.0331	153.6380	149.6330	149.8262
	EGEE	0.2487	0.0500	187.4173	193.0221	189.0173	189.2103
	NEC	0.1300	0.6902	159.4349	163.6385	160.3581	160.7797
	Chen	0.1467	0.5382	158.4795	161.2819	158.9239	159.3760
	Maxwell	0.2225	0.1023	207.0714	208.4726	207.2143	207.5197
**Dataset II**	**EGP**	0.0994	0.7770	323.1681	330.3049	324.1937	325.8148
	EGWE	0.1061	0.7046	327.5017	338.2068	329.7719	331.4716
	P	0.1319	0.4274	326.5017	330.0702	326.7945	327.8251
	EGEG	0.1062	0.7035	325.5719	332.7087	326.5975	328.2186
	NEC	0.1273	0.4732	329.6129	334.9655	330.2130	331.5979
	LOMAX	0.1568	0.2289	332.8205	336.3888	333.1132	334.1438
	Frechet	0.1771	0.1265	352.2830	355.8514	352.5757	353.6063

**Table 9:** ML estimates of EGP parameters and entropy measures based on OS and UR for Dataset I.

Parameters, Entropy Measures	OS		UR	
	Estimates (LL, UL)	S E	Estimates (LL, UL)	S E
α	1.3856 (1.3481, 1.4231)	0.0191	1.2087 (1.2083, 1.2091)	0.0002
β	1.4024 (1.3647, 1.4401)	0.0192	1.0871 (1.086, 1.0875)	0.0002
θ	0.0001 (0.0000, 0.0002)	0.00001	4.3170 (4.3160, 4.3181)	0.0006
λ	0.2059 (0.1182, 0.2936)	0.0447	0.1344 (0.1333, 0.1354)	0.0006
HS	2.3663 (2.2961, 2.4365)	0.0358	−2.1193 (−2.1528,−2.085)	0.0171
$HR_{0.3}$	2.5925 (2.5331, 2.6519)	0.0303	1.3452 (1.3407, 1.3497)	0.0022
$HR_{0.5}$	2.5278 (2.4569, 2.5986)	0.0361	0.7140 (0.7069, 0.7211)	0.0036
$HR_{0.9}$	2.3944 (2.3357, 2.4530)	0.0299	−1.2732 (−1.2991,−1.247)	0.0132
$HT_{0.3}$	7.3424 (6.9777, 7.7071)	0.18607	2.2347 (2.2231, 2.2462)	0.0058
$HT_{0.5}$	5.0784 (4.8278, 5.3291)	0.1278	0.8580 (0.8479, 0.8682)	0.0052
$H{TT}_{0.9}$	2.7054 (2.63091, 2.4365)	0.0380	−1.1954 (−1.218, −1.1726)	0.0116

**Table 10:** ML estimates of EGP parameters and entropy measures based on ROS and LR for Dataset II.

Parameters, Entropy Measures	ROS		LR	
	Estimates (LL, UL)	S E	Estimates (LL, UL)	S E
α	2.6587 (2.2152,3.1022)	0.2262	1.4061 (0.0000,2.82534)	0.7241
β	1.0001 (0.9999,1.0002)	0.0001	1.0307 (0.86025, 1.20123)	0.0869
θ	0.3000 (0.0000, 0.7997)	0.2549	0.00489 (0.0000,0.0265)	0.0110
λ	0.2038 (0.1241,0.2835)	0.0407	3.3659 (0.6694,6.0625)	1.3757
HS	3.5829 (3.34158,3.8242)	0.1231	2.5762 (1.82420,3.3282)	0.3837
$HR_{0.3}$	3.7915 (3.69520,3.8878)	0.0491	2.9650 (2.4466,3.48347)	0.2644
$HR_{0.6}$	3.7036 (3.5667,3.8404)	0.0698	2.72433 (2.0638,3.3848)	0.3370
$HR_{0.9}$	3.6132 (3.37089,3.8554)	0.1236	2.5975 (1.9644,3.23073)	0.3230
$HT_{0.3}$	18.8738 (17.5050,20.2426)	0.6983	9.9557 (5.8246,14.086)	2.1076
$HT_{0.6}$	8.4983 (3.5667,9.10039)	0.3072	4.9337 (2.96961,6.8978)	1.0020
$HT_{0.9}$	4.3521 (4.0044,4.6998)	0.1774	2.9661 (1.2589,3.7871)	0.3230

## Data Availability

All data generated or analyzed through the paper are associated with its references and sources.
